# The Selective Antagonism of Adenosine A_2B_ Receptors Reduces the Synaptic Failure and Neuronal Death Induced by Oxygen and Glucose Deprivation in Rat CA1 Hippocampus *in Vitro*

**DOI:** 10.3389/fphar.2018.00399

**Published:** 2018-04-24

**Authors:** Irene Fusco, Filippo Ugolini, Daniele Lana, Elisabetta Coppi, Ilaria Dettori, Lisa Gaviano, Daniele Nosi, Federica Cherchi, Felicita Pedata, Maria G. Giovannini, Anna M. Pugliese

**Affiliations:** ^1^Department of Neuroscience, Psychology, Drug Research and Child Health, NEUROFARBA, Section of Pharmacology and Toxicology, University of Florence, Florence, Italy; ^2^Department of Health Sciences, Section of Clinical Pharmacology and Oncology, University of Florence, Florence, Italy; ^3^Department of Experimental and Clinical Medicine, University of Florence, Florence, Italy

**Keywords:** apoptosis, MRS1754, PSB603, OGD, anoxic depolarization, mTOR, confocal microscopy, neurodegeneration

## Abstract

Ischemia is a multifactorial pathology characterized by different events evolving in time. Immediately after the ischemic insult, primary brain damage is due to the massive increase of extracellular glutamate. Adenosine in the brain increases dramatically during ischemia in concentrations able to stimulate all its receptors, A_1_, A_2A_, A_2B_, and A_3_. Although adenosine exerts clear neuroprotective effects through A_1_ receptors during ischemia, the use of selective A_1_ receptor agonists is hampered by their undesirable peripheral side effects. So far, no evidence is available on the involvement of adenosine A_2B_ receptors in cerebral ischemia. This study explored the role of adenosine A_2B_ receptors on synaptic and cellular responses during oxygen and glucose deprivation (OGD) in the CA1 region of rat hippocampus *in vitro*. We conducted extracellular recordings of CA1 field excitatory post-synaptic potentials (fEPSPs); the extent of damage on neurons and glia was assessed by immunohistochemistry. Seven min OGD induced anoxic depolarization (AD) in all hippocampal slices tested and completely abolished fEPSPs that did not recover after return to normoxic condition. Seven minutes OGD was applied in the presence of the selective adenosine A_2B_ receptor antagonists MRS1754 (500 nM) or PSB603 (50 nM), separately administered 15 min before, during and 5 min after OGD. Both antagonists were able to prevent or delay the appearance of AD and to modify synaptic responses after OGD, allowing significant recovery of neurotransmission. Adenosine A_2B_ receptor antagonism also counteracted the reduction of neuronal density in CA1 stratum pyramidale, decreased apoptosis at least up to 3 h after the end of OGD, and maintained activated mTOR levels similar to those of controls, thus sparing neurons from the degenerative effects caused by the simil-ischemic conditions. Astrocytes significantly proliferated in CA1 stratum radiatum already 3 h after the end of OGD, possibly due to increased glutamate release. A_2B_receptor antagonism significantly prevented astrocyte modifications. Both A_2B_ receptor antagonists did not protect CA1 neurons from the neurodegeneration induced by glutamate application, indicating that the antagonistic effect is upstream of glutamate release. The selective antagonists of the adenosine A_2B_ receptor subtype may thus represent a new class of neuroprotective drugs in ischemia.

## Introduction

Cerebral ischemic stroke represents a life threatening neurological disorder that leads to mortality and long-term disability in surviving patients. Ischemic stroke remains one of the main causes of death and disability in the western countries with only very limited therapeutic options (Dirnagl, 2012).

Acute brain injury after stroke is caused primarily by the lack of oxygen and glucose. In such conditions, mammalian neurons rapidly depolarize, and excessive release of glutamate occurs, causing excitotoxic cell death, largely due to over-activation of glutamatergic *N*-methyl-D-aspartate (NMDA) receptors. NMDA receptors are highly permeable to Ca^2+^ and are responsible for intracellular Ca^2+^ increase that reaches neurotoxic levels which, by activating cell lipases, endonucleases, proteases, and phosphatases, ultimately bring to acute excitotoxic cell death (Choi, 1992). Also, one of the early events occurring by an ischemic episode *in vivo* and during oxygen and glucose deprivation (OGD) *in vitro*, is the release of substantial amounts of adenosine (Latini et al., 1998; Melani et al., 1999; Frenguelli et al., 2007).

Adenosine exerts its biological functions via four receptors subtypes, A_1_, A_2A_, A_2B_, and A_3_ (Latini and Pedata, 2001). Many studies indicate that A_1_ receptors play a prominent inhibitory tone on synaptic transmission and that adenosine selective antagonists, acting on this receptor subtype, has a protective role under ischemia (Pedata et al., 2016). Unfortunately, the development of A_1_ receptor selective agonists as possible anti-ischemic drugs has been stalled by their sedative and cardiovascular side effects, including bradycardia and hypotension. Therefore, in order to identify putative targets for therapeutic intervention, the research on possible anti-ischemic drugs has focussed on the contribution of the other adenosine receptors. The role of the adenosine A_2A_ receptor under ischemia has been largely investigated (Chen et al., 2007; Pedata et al., 2014). Among adenosine receptors, the A_2B_ receptor subtype is the least studied and still remains the most enigmatic, because of the relatively low potency of adenosine for this receptor (Fredholm et al., 2011) and the very few selective ligands that have been described so far. Most of the present knowledge on A_2B_ receptors originates from their peripheral role on the control of cardiac myocyte contractility, intestinal tone, asthma, inflammation, cancer and diabetes (Feoktistov et al., 1998; Kolachala et al., 2008; Chandrasekera et al., 2010; Merighi et al., 2015; Allard et al., 2017). A_2B_ receptors play proinflammatory roles in human asthma, in chronic obstructive pulmonary disease and murine colitis (Feoktistov et al., 1998; Csóka et al., 2007; Kolachala et al., 2008). In the central nervous system (CNS), adenosine A_2B_ receptors, although scarcely, are uniformly expressed (Dixon et al., 1996) including in the hippocampus (Perez-Buira et al., 2007), but their role or function and in particular under ischemic/hypoxic conditions is still to be clarified. Understanding the processes by which the applications of these compounds confer neuroprotection should shed light on mechanisms to delay or mitigate the pathophysiological effects of ischemic injury.

In this paper we investigated the role of adenosine A_2B_ receptors during OGD in the CA1 region of rat hippocampus, the most susceptible hippocampal area to an ischemic insult. For this purpose two selective adenosine A_2B_ receptor antagonists were used. In order to characterize the OGD-induced cell injury and putative pharmacological protection, we conducted extracellular recordings of CA1 field excitatory post-synaptic potentials (fEPSPs) after a severe (7 min or 30 min) simil-ischemic insult. The response to ischemia consists of complex, concerted actions of the CNS and the peripheral immune system, that is very difficult to reproduce in *in vitro* model. However, these OGD episodes bring about irreversible depression of neurotransmission and the appearance of anoxic depolarization (AD) (Frenguelli et al., 2007; Pugliese et al., 2007). AD is a severe neuronal depolarization, which is an early and critical event that has been demonstrated both *in vivo* (Somjen, 2001) and *in vitro* (Tanaka et al., 1997; Pugliese et al., 2006). AD triggers a variety of molecular events, contributes to cell death and represents an unequivocal sign of neuronal injury (Somjen, 2001). The amount of time spent by neurons in AD is an important determinant of neuron fate. Propagation of AD from the ischemic core is one major factor contributing to neuronal death in the area surrounding the ischemic core (the penumbra) (Koroleva and Bures, 1996). The penumbra constitutes potentially salvageable tissue and hence a pharmacological treatment that delays the onset of AD would help to protect brain tissue from ischemia (Jarvis et al., 2001; Somjen, 2001).

Cell viability, extent of neuronal damage, astrocytes immunoreactivity and activation of apoptosis markers were also assessed by immunohistochemical analysis. Preliminary data were presented at the Society for Neuroscience Meeting (Ugolini et al., 2017).

## Materials and Methods

All animal experiments were performed according to the Italian Law on Animal Welfare (DL 26/2014), approved by the Institutional Animal Care and Use Committee of the University of Florence and by the Italian Ministry of Health. All efforts were made to minimize animal sufferings and to use only the number of animals necessary to produce reliable scientific data. Male Wistar rats (Envigo, Italy, 150–200 g body weight) were used. Experiments were carried out on acute rat hippocampal slices, prepared as previously described (Pugliese et al., 2006, 2009).

### Preparation of Slices

Animals were killed with a guillotine under anesthesia with isoflurane (Baxter, Rome, Italy) and hippocampi were rapidly removed and placed in ice-cold oxygenated (95% O_2_–5% CO_2_) artificial cerebrospinal fluid (aCSF) of the following composition (mM): NaCl 124, KCl 3.33, KH_2_PO_4_ 1.25, MgSO_4_ 1.4, CaCl_2_ 2.5, NaHCO_3_ 25, and D-glucose 10. Slices (400 μm nominal thickness) were cut using a McIlwain Tissue Chopper (Mickle Laboratory Engineering Co. Ltd., Gomshall, United Kingdom) and kept in oxygenated aCSF for at least 1 h at room temperature. A single slice was then placed on a nylon mesh, completely submerged in a small chamber (0.8 ml) and superfused with oxygenated aCSF (31–32°C) at a constant flow rate of 1.5 ml/min. The treated solutions reached the preparation in 60 s and this delay was taken into account in our calculations.

### Extracellular Recordings

**FIGURE 1:**
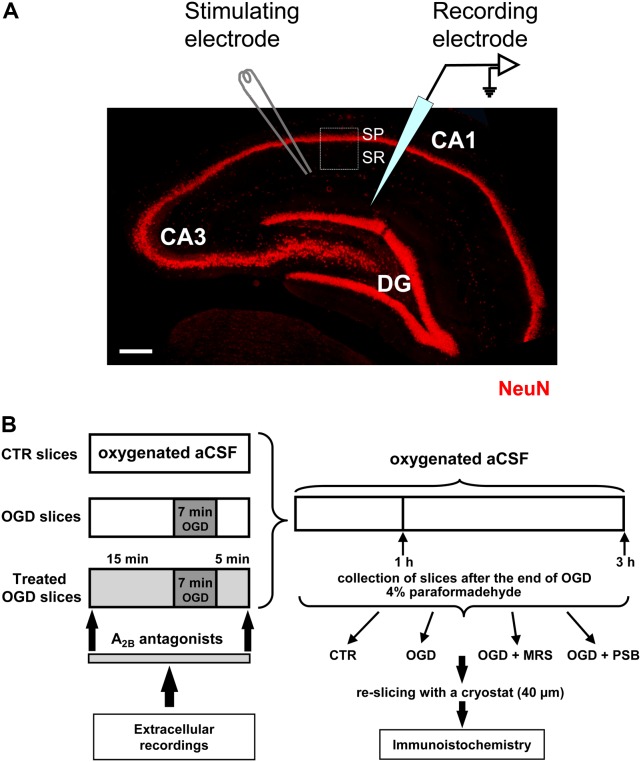
Experimental methods. **(A)** Microphotography of an hippocampal slice showing the three subregions, the localization of the stimulating and recording electrodes and the region of interest (ROI, framed area) for the immunohistochemical analyses. SP, stratum pyramidale; SR, stratum radiatum. Scale bar: 200 μm. **(B)** Schematic representation of the experimental method.

Test pulses (80 μs, 0.066 Hz) were delivered through a bipolar nichrome electrode positioned in the stratum radiatum of the CA1 region of the hippocampus to stimulate the Schaffer collateral-commissural pathway (**Figure 1A**). Evoked potentials were extracellularly recorded with glass microelectrodes (2–10 MΩ, Harvard Apparatus LTD, United Kingdom) filled with 150 mM NaCl. The recording electrode was placed at the dendritic level of the CA1 region to record field excitatory postsynaptic potentials (fEPSPs) (**Figure 1A**). Responses were amplified (200×, BM 622, Mangoni, Pisa, Italy), digitized (sample rate, 33.33 kHz), and stored for later analysis with LTP (version 2.30D) program (Anderson and Collingridge, 2001). The amplitude of fEPSP was measured as the difference between the negative peak following the afferent fiber volley and the baseline value preceding the stimulus artifact. In some experiments both the amplitude and the initial slope of fEPSP were quantified, but since no appreciable difference between these two parameters was observed under control conditions, in the presence of drugs or during *in vitro* ischemia, only the measure of the amplitude was expressed in the figures. When a stable baseline of evoked responses was reached, fEPSP amplitudes were routinely measured and expressed as the percentage of the mean value recorded 5 min before the application of any treatment (in particular pre-OGD). Stimulus-response curves were obtained by gradual increase in stimulus strength at the beginning of each experiment. The test stimulus strength was then adjusted to produce a response whose amplitude was 40% of the maximum and was kept constant throughout the experiment. Simultaneously, with fEPSP amplitude, AD was recorded as negative extracellular direct current (d.c.) shifts induced by OGD. The d.c. potential is an extracellular recording considered to provide an index of the polarization of cells surrounding the tip of the glass electrode (Farkas et al., 2008). AD latency, expressed in min, was calculated from the beginning of OGD; AD amplitude, expressed in mV, was calculated at the maximal negativity peak. In the text and bar graphs, AD amplitude values were expressed as positive values. The terms “irreversible synaptic failure” or “irreversible loss of synaptic transmission” used in the present work refer to the maximal time window of cell viability in our experimental model (acutely isolated hippocampal slice preparation) which, according to our previous results is 24 h (Pugliese et al., 2009).

#### Paired-Pulse Facilitation

To elicit paired-pulse facilitation (PPF) of fEPSP, we stimulated the Schaffer collateral-commissural fibers twice with a 40-ms interpulse interval. Double stimulation was evoked once every 15 s. The synaptic facilitation was quantified as the ratio (P2/P1) between the slope of the fEPSP elicited by the second (P2) and the first (P1) stimuli. PPF was monitored in control conditions for at least 5 min before the application of BAY606583. The effect of BAY606583 on PPF was evaluated by measuring the P2/P1 ratio during at least 5 min after 15 min of agonist application.

### Drugs

Two selective adenosine A_2B_ receptors antagonists, N-(4-Cyanophenyl)-2-[4-(2,3,6,7-tetrahydro-2,6-dioxo-1,3-dip-ropyl-1H-purin-8-yl)phenoxy]-acetamide) (MRS1754) and 8-[4-[4-(4-Chlorophenzyl) piperazide-1-sulfonyl) phenyl]]-1-propyl xanthine (PSB603) were used. D-2-amino-5-phosphonovalerate, a selective NMDA receptor antagonist was used. All these compounds were purchased from Tocris (Bristol, United Kingdom). The A_1_ receptor antagonist DPCPX (8-cyclopentyl-1,3-dipropylxanthine) was purchased from SIGMA Aldrich (https://www.sigmaaldrich.com).

All drugs were dissolved in dimethyl sulphoxide (DMSO). Stock solutions, of 1000–10,000 times the desired final concentration, were stored at -20°C. The final concentration of DMSO (0.05% and 0.1% in aCSF) used in our experiments did not affect either fEPSP amplitude or the depression of synaptic potentials induced by OGD (data not shown).

### Application of OGD and Adenosine A_2B_ Receptor Antagonists

The experimental method is shown in **Figure 1B**. Conditions of OGD were obtained by superfusing the slice with aCSF without glucose and gassed with nitrogen (95% N_2_–5% CO_2_) (Pedata et al., 1993). This causes a drop in pO_2_ in the recording chamber from ∼500 mmHg (normoxia) to a range of 35–75 mmHg (after 7 min OGD) (Pugliese et al., 2003). At the end of the ischemic period, the slice was again superfused with normal, glucose-containing, oxygenated aCSF. The terms ‘OGD slices’ or ‘treated OGD slices’ refer to hippocampal slices in which OGD was applied in the absence or in the presence of A_2B_ receptor antagonists, respectively. Control slices were not subjected to OGD or treatment with A_2B_ receptor antagonists but were incubated in oxygenated aCSF for identical time intervals. All the selective adenosine A_2B_ receptors antagonists were applied 15 min before, during and 5 min after OGD. In a typical experimental day, first a control slice was subjected to 7 min of OGD. If the recovery of fEPSP amplitude after 60 min of reperfusion with glucose containing and normally oxygenated aCSF was ≤15% of the pre-OGD value, and AD developed into 7 min OGD, a second slice from the same rat was subjected to an OGD insult in the presence of the A_2B_ receptor antagonist under investigation. To confirm the result obtained in the treated group, a third slice was taken from the same rat and another 7 min OGD was performed under control conditions to verify that no difference between slices was caused by the time gap between the experiments. In some slices the OGD period was prolonged to 30 min and the A_2B_ receptor antagonists were applied 15 min before and during OGD application. After the extracellular recordings, slices were maintained in separate chambers for 1 or 3 h from the end of OGD in oxygenated aCSF at room temperature (RT). At the end, slices were harvested and fixed overnight at 4°C in 4% paraformaldehyde in PBS, cryopreserved in 18% sucrose for 48 h, and resliced as written below.

### Treatment of Hippocampal Slices With Glutamate *in Vitro*

Experiments were carried out on acute hippocampal slices, prepared from male Wistar rats as described above. The A_2B_ receptor antagonists were dissolved in DMSO to obtain a stock solution suitable for a 1:2000 dilution. Slices, maintained oxygenated throughout the procedure, were incubated according to the following scheme:

• Control slices were incubated for 1 h in aCSF and then for 25 min in aCSF with DMSO (1:2000; 0.05%);
• Glutamate (GLU) treated slices were incubated 1 h in aCSF and then for 10 min with 100 μM glutamate in aCSF;
• MRS+GLU treated slices were incubated for 1 h in aCSF, then for 15 min with 500 nM MRS1754 and for further 10 min with 500 nM MRS1754 plus 100 μM glutamate, in aCSF;
• PSB+GLU treated slices were incubated for 1 h in aCSF, then for 15 min with 50 nM PSB603, and for further 15 min with 50 nM PSB603 plus 100 μM glutamate in aCSF;

After the incubation with glutamate and A_2B_ receptor antagonists, slices were further incubated for 3 h in aCSF, and then harvested and fixed overnight at 4°C in 4% paraformaldehyde in PBS, cryopreserved in 18% sucrose for 48 h, and resliced as written below.

### Immunohistochemistry

One hour or 3 h after OGD, or after the incubation with glutamate and A_2B_ receptor antagonists, the 400 μm thick slices fixed in paraformaldehyde were placed on an agar support (6% agar in normal saline), included in an embedding matrix and re-sliced with a cryostat to obtain 40 μm thick slices. The more superficial sections were eliminated, while those obtained from the inner part of the slice were collected and stored in vials with 1 ml of antifreeze solution at -20°C until immunohistochemical analyses. From the 400 μm thick slices on average only a maximum of 2–3 complete 40 μm thick slices were obtained, which were then randomly allocated to the fluorescent immunohistochemical staining groups.

#### Antibodies Used – Primary Antibodies

Neurons were immunostained with a mouse monoclonal anti-NeuN antibody (1:200, MilliporeSigma, Carlsbad, CA, United States), astrocytes were detected by means of a polyclonal rabbit antibody anti-Glial Fibrillary Acidic Protein (GFAP, 1:500, DakoCytomation, Glostrup, Denmark), Cytochrome C with a mouse monoclonal antibody (1:200, Abcam, Cambridge, United Kingdom). Activated mTOR was detected using a polyclonal rabbit primary antibody raised against phospho-(Ser2448)-mTOR (1:100, Abcam, Cambridge, United Kingdom). Fluorescent secondary antibodies: Alexa Fluor 488 donkey anti rabbit (fluorescence in green, 1:400), Alexa Fluor 555 donkey anti mouse (fluorescence in red, 1:400), Alexa Fluor 635 goat anti-rabbit (fluorescence in far red, 1:400) (all from Life Technologies, Carlsbad, CA, United States). All primary and secondary antibodies were dissolved in Blocking Buffer (BB, 10% Normal Goat Serum, 0.05% NaN_3_ in PBS-TX). All procedures were carried out with the free-floating method in wells of a 24-well plate (Cerbai et al., 2012; Lana et al., 2013).

#### Day 1

The sections were washed (3 times, 5 min each) in PBS-0.3% Triton X-100 (PBS-TX), blocked with 500 μl BB for 1 h, at RT under slight agitation and then incubated overnight at 4°C with the primary antibody under slight agitation.

#### Day 2

After washing in PBS-TX (3 times, 5 min each), sections were incubated for 2 h at room temperature in the dark with a solution containing one or two (for double immunostaining) fluorescent secondary antibodies, as appropriate. Sections were washed (3 times, 5 min each) with BB and then with 1 ml of distilled H_2_O at RT in the dark, mounted on gelatinized microscopy slides, dried and coverslipped with a mounting medium containing DAPI to counterstain nuclei (Vectashield, Hard set mounting medium with DAPI, Vector Laboratories, Burlingame, CA, United States). Sections were kept refrigerated in the dark until microscopy analyses.

#### Day 3

Qualitative and quantitative analyses of NeuN positive neurons, CytC and phospho-mTOR positive cell bodies were performed in CA1 stratum pyramidale (SP), while astrocytes, phospho-mTOR positive dendrites and microglia were performed in CA1 stratum radiatum (SR) as shown in **Figure 1A**. Epifluorescence microscopy: sections were observed under an Olympus BX63 microscope equipped with an Olympus DP 50 digital camera (Olympus, Milan, Italy). For quantitative analysis images were acquired at 20× magnification with the digital camera.

#### Confocal Microscopy

Scans were taken at 0.3 μm z-step, keeping constant all the parameters (pinhole, contrast, and brightness), using a LEICA TCS SP5 confocal laser scanning microscope (Leica Microsystems CMS GmbH, Mannheim, Germany). Images were converted to green, or red using ImageJ (freeware provided by National Institute of Health^1^). The region of interest (ROI) in CA1, containing stratum pyramidalis and stratum radiatum was consistently analyzed in all slices, as shown in **Figure 1A** (Lana et al., 2014). Quantitative analyses of NeuN^+^ neurons, HDN neurons, LDN neurons, GFAP^+^ astrocytes, CytC^+^ apoptotic neurons and phospho-mTOR positive cell bodies and dendrites were performed blind by two experimenters and results were averaged. Areas were expresses as mm^2^. Digitized images were transformed into TIFF files and thresholded using ImageJ. Care was taken to maintain the same threshold in all sections within the same experiment. In CA1 pyramidal layer, the area labeled above the set threshold with NeuN or phospho-mTOR was calculated in pixels and expressed as NeuN^+^ pixels/mm^2^ or phospho-mTOR^+^ pixels/mm^2^. HDN neurons, LDN neurons, Cytochrome C-positive (CytC^+^) apoptotic neurons in CA1 stratum pyramidale and GFAP^+^ astrocytes in CA1 stratum radiatum were counted and were expressed as number of cells/mm^2^. In order to evaluate mTOR activation in basal dendrites the length of phospho-mTOR^+^ dendrites was measured at three fixed locations, equal in all slices and evenly distributed throughout the CA1 stratum radiatum ROI, and results were averaged.

### Statistical Analysis

Statistical significance was evaluated by Student’s paired or unpaired *t-*tests. Analysis of variance (one-way ANOVA), followed by Newman–Keuls multiple comparison *post hoc* test was used, as appropriate. *P*-values from both Student’s paired and unpaired *t*-tests are two-tailed. Data were analyzed using software package GraphPad Prism (version 7.0; GraphPad Software, San Diego, CA, United States). All numerical data are expressed as the mean ± standard error of the mean (SEM). A value of *P* < 0.05 was considered significant.

## Results

### Electrophysiological Experiments

It has been established that 7 min OGD episodes bring about irreversible depression of neurotransmission and the appearance of a severe neuronal depolarization or AD (Pugliese et al., 2006, 2007, 2009), a critical event that has been demonstrated both *in vivo* (Somjen, 2001) and *in vitro* (Fowler, 1992; Pearson et al., 2006; Pugliese et al., 2006, 2007, 2009; Frenguelli et al., 2007). Therefore, we studied the effects of two selective adenosine A_2B_ receptor antagonists, MRS1754 and PSB603, on AD development in the CA1 region of acute rat hippocampal slices under severe OGD episodes by extracellular recording of fEPSPs on 133 hippocampal slices taken from 42 rats.

### The Selective Adenosine A_2B_ Receptor Antagonism Prevents or Delays AD Development and Protects From Synaptic Failure Induced by Severe OGD in CA1 Hippocampus

**FIGURE 2:**
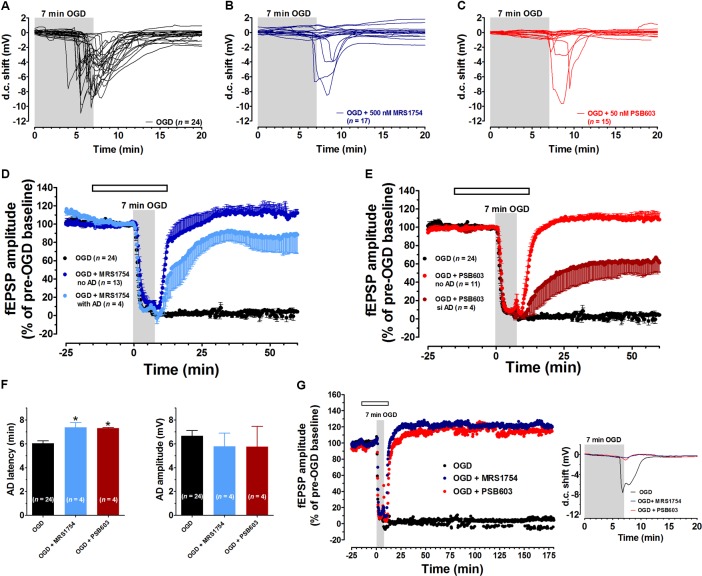
The selective adenosine A_2B_ receptor antagonists MRS1754 or PSB603 significantly reduced the synaptic failure induced by 7 min oxygen and glucose deprivation (OGD) in the CA1 region of rat hippocampal slices. **(A–C)** anoxic depolarization (AD) was recorded as a negative direct current (d.c.) shift in response to 7 min OGD in untreated OGD slices **(A)**, in 500 nM MRS1754-treated slices **(B),** or 50 nM PSB603-treated slices **(C)**. Note that MRS1754 prevented the appearance of AD in 13 out of 17 slices, while PSB603 in 11 out of 15 slices. **(D)** The graph shows the time-course of the effect of 7 min OGD on field excitatory post-synaptic potential (fEPSP) amplitude, expressed as percentage of pre-OGD baseline in the CA1 hippocampal region in the absence (*n* = 24) or in the presence of 500 nM MRS1754 (*n* = 17). Note that, in untreated slices, the ischemic-like insult caused gradual reduction, up to disappearance, of fEPSPs amplitude that did not recover after washing in oxygenated artificial cerebrospinal fluid (aCSF). On the contrary, after reperfusion in oxygenated standard solution, a recovery of fEPSP in all MRS1754 treated OGD slices was found, even in those in which AD developed. **(E)** The graph shows the time course of the effect of 7 min OGD on fEPSP amplitude in 50 nM PSB603 treated OGD slices. Note that, after reperfusion in normal oxygenated standard solution, a recovery of fEPSP was found in all OGD-treated PSB603 slices, even those in which AD occurred. **(F, Left)** each column represents the mean ± SEM of AD latency recorded in the CA1 region during 7 min OGD in the absence or in the presence of MRS1754 (500 nM) or PSB603 (50 nM). AD latency was measured from the beginning of OGD insult. Note that when OGD was applied in the presence of MRS1754 or PSB603 the appearance of AD was significantly delayed in comparison to OGD untreated slices. ^∗^*P* < 0.05 vs. OGD, One-way ANOVA followed by Newman–Keuls Multiple comparison test. **(Right)** each column represents the mean ± SEM of AD amplitude recorded in the CA1 during 7 min OGD. The number of slices is reported in the columns. **(G)** The graph shows the time course of the effect of 7 min OGD on fEPSP amplitude in OGD-untreated slices and in 500 nM MRS1754- or 50 nM PSB603-treated slices. The selective antagonism of adenosine A_2B_ receptors counteracted the CA1 synaptic damage induced by severe OGD up to 3 h from the end of the insult. Inset: 7 min OGD induced AD was recorded untreated OGD slices, but not in the presence of 500 nM MRS1754 or 50 nM PSB603. Gray bar: OGD time duration. Open bar: time of drug application. Amplitude of fEPSPs (mean ± SEM) is expressed as percentage of pre-OGD baseline.

In agreement with our previous results (Pugliese et al., 2006, 2007, 2009), in untreated OGD slices the d.c. shift presented a mean latency of 6.04 ± 0.2 min (calculated from the beginning of OGD) and a mean peak amplitude of -6.7 ± 0.4 mV (*n* = 24) (**Figure 2A**). Seven min OGD exposure induced a rapid and irreversible depression of fEPSPs amplitude evoked by Schaffer-collateral stimulation, since synaptic potentials did not recover their amplitude after return to oxygenated aCSF (**Figure 2D**, *n* = 24, 2.5 ± 2.7% of pre-OGD level, calculated 50 min from the end of OGD). Control slices, followed for up to 3 h in oxygenated aCSF, maintained stable fEPSPs for the entire experimental time recording and never developed the d.c. shift (data not shown).

Oxygen and glucose deprivation was then applied in the presence of the selective adenosine A_2B_ receptor antagonists MRS1754 or PSB603, administered 15 min before, during and 5 min after OGD.

The two A_2B_ receptor antagonists did not modify basal synaptic transmission measured before OGD. Indeed, MRS1754 (500 nM, *n* = 17) did not modify fEPSPs amplitude under normoxic conditions (from 1.05 ± 0.06 mV immediately before to 1.01 ± 0.08 mV after 15 min drug application, *n* = 17). Also, PSB603 did not change the amplitude of synaptic potentials under normoxic conditions (from 1.32 ± 0.12 mV before to 1.35 ± 0.14 mV after 15 min drug application, *n* = 15). These data indicate that the blockade of A_2B_ receptors does not modify low-frequency-induced CA1 synaptic transmission under normoxic conditions, in agreement with results reported in mouse hippocampal slices (Gonçalves et al., 2015).

Nevertheless, the two A_2B_ receptor antagonists were able to prevent or delay the appearance of AD and to modify synaptic responses after OGD.

During 7 min OGD, MRS1754 prevented the appearance of AD in 13 out of 17 slices tested (**Figure 2B**). In these 13 slices a complete recovery of fEPSPs was recorded (111.9 ± 7.4%, calculated 50 min from the end of OGD, **Figure 2D**). In the remaining 4 slices, AD developed, although at later times (**Figure 2F**, mean AD latency: 7.37 ± 0.41 min; mean peak amplitude: -5.8 ± 1.1 mV, *n* = 4), and, unexpectedly, was followed by a consistent fEPSP recovery (85.2 ± 15.3%, *n* = 4, **Figure 2D**).

During 7 min OGD, PSB603 prevented the appearance of AD in 11 out of 15 slices tested (**Figure 2C**). In these 11 slices a complete recovery of fEPSPs was found (110.4 ± 10.2%, *n* = 11, **Figure 2E**). In the remaining four slices in which AD appeared, a delay in AD latency was recorded (**Figure 2F**, mean AD latency: 7.33 ± 0.08 min; mean peak amplitude: -6.8 ± 1.9 mV, *n* = 4). Moreover, in these four PSB603-treated slices, a significant recovery of fEPSP (36.2 ± 19.7%, *n* = 4, **Figure 2E**) was found.

In the slices in which AD appeared in the presence of MRS1754 or PSB603, we compared the time of AD appearance in the absence and in the presence of drugs. As illustrated in **Figure 2F**, during 7 min OGD, AD appeared in OGD slices with a mean latency of 6.04 ± 0.2 min (Left panel) and a mean peak amplitude of 6.7 ± 0.4 mV (*n* = 24, Right panel). When 7 min OGD was applied in the presence of 500 nM MRS1754 or 50 nM PSB603 the d.c. shifts were always delayed (**Figure 2F**, Left panel), while AD amplitude values were not significantly modified in comparison to OGD slices (**Figure 2F**, Right panel).

In an experimental group of slices which never developed AD in the presence of PSB603 (50 nM) (*n* = 6) and MRS1754 (500 nM) (*n* = 6), we followed the evolution of the synaptic response for 3 h after the end of the 7 min ischemic like insult in comparison to untreated OGD slices (*n* = 6). As reported in the representative electrophysiological traces shown in **Figure 2G**, PSB603 (50 nM) and MRS1754 (500 nM) allowed the recovery of synaptic potentials for at least 3 h after 7 min OGD.

Furthermore, in order to confirm that both the recovery of fEPSP and the irreversible loss of neurotransmission after 7 min OGD observed in the different experimental groups were not transient, we tested slice viability 24 h after the OGD insult in control, untreated, slices and slices treated with A_2B_-receptor antagonists. In agreement with our previously published results (Pugliese et al., 2009), we showed that untreated OGD slices, which did not recover any synaptic activity within 1 h after the insult, maintained synaptic impairment when tested 24 h later (Supplementary Figure 1A shows a representative experiment out of a total of six slices). On the contrary, MRS1754- or PSB603-treated OGD slices, which recovered initial fEPSP amplitude 1 h after OGD, preserved neurotransmission for at least 24 h after the insult (Supplementary Figure 1B shows a representative experiment out of a total of four slices, and Supplementary Figure 1C shows a representative experiment out of a total of five slices for MRS1754 and PSB603, respectively).

**FIGURE 3:**
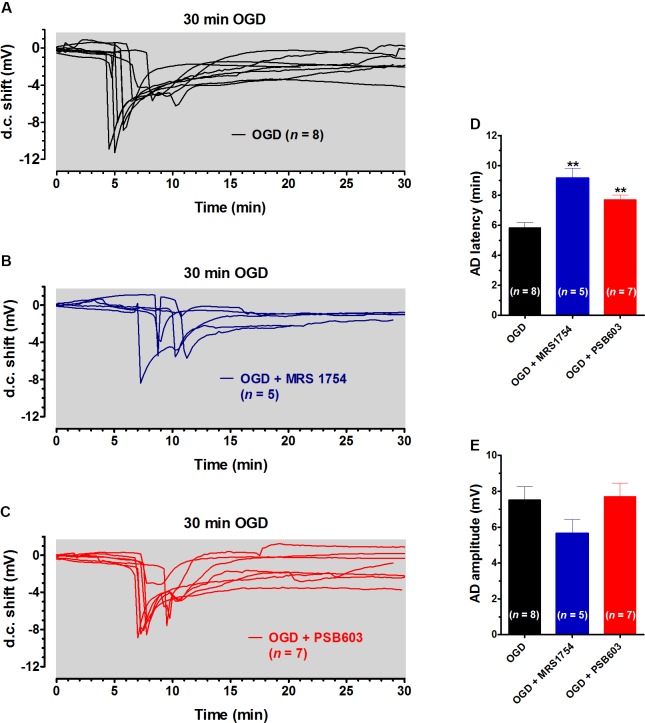
MRS1754 and PSB603 delayed the appearance of AD induced by 30 min OGD in rat hippocampal slices. **(A–C)** The graphs show the d.c. shift traces during 30 min OGD in untreated OGD slices (**A**, *n* = 8), in the presence of 500 nM MRS1754 (**B**, *n* = 5), or 50 nM PSB603 (**C**, *n* = 7). **(D)** Each column represents the mean ± SEM of AD latency recorded in hippocampal slices during 30 min OGD in different experimental groups. AD was measured from the beginning of OGD insult. Note that both adenosine A_2B_ receptor antagonists significantly delayed AD development. ^∗∗^*P* < 0.01 vs. OGD, One-way ANOVA followed by Newman–Keuls Multiple comparison test. **(E)** Each column represents the mean ± SEM of AD amplitude recorded in the CA1 during 30 min OGD. The number of slices is reported in the columns.

In order to characterize the role of adenosine A_2B_ receptors on AD development, in a next series of experiments we prolonged the OGD duration up to 30 min, in order to allow AD to unavoidably appear in all experimental groups. This longer duration of OGD is invariably associated with tissue damage (Pearson et al., 2006). We compared the latency and the magnitude of depolarizing d.c. shifts recorded in the absence or presence of PSB603 or MRS1754. As illustrated in **Figure 3A**, 30 min OGD elicited the appearance of AD in all slices, with a mean peak amplitude of -7.5 ± 0.7 mV (*n* = 8) and a mean latency of 5.8 ± 0.3 min, as shown in **Figures 3D,E**. When OGD was applied in the presence of 500 nM MRS1754, the d.c. shift was significantly delayed to 9.2 ± 0.7 min (**Figures 3B, D**; *n* = 5), although the AD amplitude (-5.7 ± 0.7 mV) was not significantly changed (**Figure 3E**). Similarly, when OGD was applied in the presence of 50 nM PSB603, the d.c. shift was significantly delayed to 7.7 ± 0.3 min (**Figures 3C,D**; *n* = 7) whereas AD amplitude (-7.7 ± 0.7 mV) was unchanged (**Figure 3E**).

Data in the literature demonstrate that adenosine A_2B_ receptors exert their effects through a control of A_1_ receptor function in the hippocampus under simil-physiological, normoxic, conditions (Gonçalves et al., 2015). In order to test this possibility, we studied whether A_2B_ receptor antagonists were still effective in inhibiting OGD-induced alterations of synaptic transmission in the presence of the A_1_ receptor antagonist DPCPX. As shown in Supplementary Figures 2A–C, we applied DPCPX before, during and after a 7 min OGD and, unexpectedly, we found that 2 out of 6 slices tested did not undergo AD and completely recovered their synaptic activity. This unexpected result was possibly due to the unselective block of A_2A_ receptors by DPCPX, as already described in hippocampal slices during OGD (Sperlágh et al., 2007). Therefore, we reduced DPCPX concentration to 100 nM and we prolonged the OGD period up to 30 min (Supplementary Figure 2D). Under these experimental conditions, DPCPX-exposed slices presented a delayed AD appearance (mean AD peak time = 8.4 ± 0.5 min) in comparison to control, untreated, OGD slices (mean AD peak time = 6.8 ± 0.2 min) thus confirming that DPCPX protects hippocampal slices from OGD insults (Supplementary Figure 2E). The time window of A_2B_ or A_1_ receptor-mediated effects found in the present studies overlaps with the delay found treating the slices with glutamate receptor antagonists (Tanaka et al., 1997; Yamamoto et al., 1997), or blocking NMDA receptors that are involved both in initiation and propagation of AD (Herreras and Somjen, 1993; Somjen, 2001). In a further series of experiments, we demonstrated that D-AP5 (50 μM) significantly delayed AD appearance (from 6.8 ± 0.2 min in untreated OGD slices to 9.8 ± 1.0 min in D-AP5-treated OGD slices, Supplementary Figure 2E).

**FIGURE 4:**
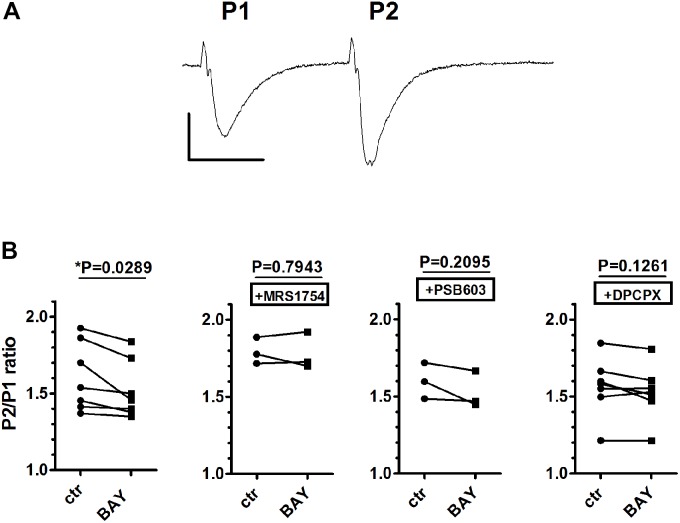
The selective stimulation of adenosine A_2B_ receptors reduced paired-pulse facilitation (PPF) in rat hippocampal slices. **(A)** Trace of fEPSP responses to PPF protocol (40-ms interval), taken from a typical experiment. Calibration: 0.5 mV, 20 ms. **(B)** Each graph shows PPF quantified as the ratio (P2/P1) between the slope of the second fEPSP (P2) and the slope first fEPSP (P1). The effect of BAY606583 (BAY, 200 nM) on PPF was investigated in the absence (*n* = 7) or in the presence of MRS1754 (500 nM, *n* = 3), PSB603 (50 nM, *n* = 3), or DPCPX (100 nM, *n* = 7). ^∗^*P* < 0.05 vs. ctr, paired Student’s *t*-test.

For this reason, in order to assess the involvement of A_1_ receptors in A_2B_ receptor-mediated effects, we choose a different protocol. It has been shown that short-term plasticity, measured by PPF, is modified by A_2B_ receptor activation in mouse hippocampal slices in a DPCPX-sensitive manner (Gonçalves et al., 2015). We confirmed that the A_2B_ receptor agonist BAY606583, at 200 nM concentration, significantly decreased PPF in rat CA1 hippocampus (**Figure 4**), thus indicating an increase of presynaptic glutamate release upon A_2B_ receptor activation. This effect was blocked not only by the A_2B_ receptor antagonists PSB603 and MRS1754, but also by the A_1_ receptor antagonist DPCPX (**Figure 4B**), thus confirming that A_2B_ receptor effects are mediated by the inhibition of the A_1_ subtype, as already stated by Gonçalves et al. (2015).

### Analysis of Neuronal Damage in CA1 Stratum Pyramidale 1 and 3 h After the End of 7 min OGD

**FIGURE 5:**
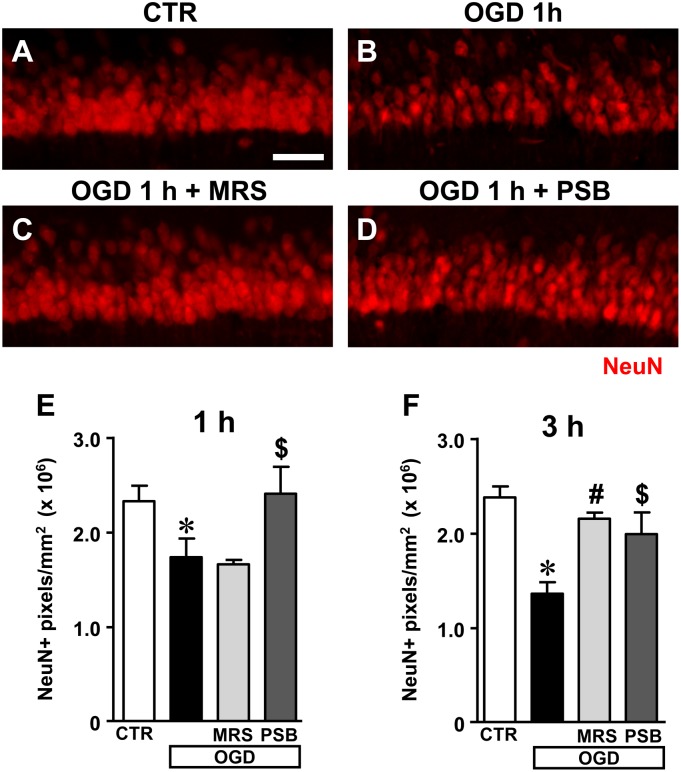
Analysis of NeuN^+^ immunofluorescence in CA1 stratum pyramidale after the simil-ischemic insult. **(A–D)** Representative images of NeuN^+^ immunofluorescence in the ROI of CA1 of a control slice (CTR, **A**), a slice collected 1 h after 7 min OGD (OGD, **B**), a slice treated with 500 nM MRS1754 (OGD+MRS, **C**), and a slice treated with 50 nM PSB603 (OGD+PSB, **D**), all harvested 1 h after the end of 7 min OGD. Scale bar: 75 μm. **(E,F)** Quantitative analyses of NeuN^+^ immunofluorescence in the four experimental groups 1 h **(E)** and 3 h **(F)** after the end of 7 min OGD. Each column represents the area, expressed in pixels (×10^6^) above a threshold, maintained constant for all slices investigated. **(E)** Statistical analysis: One-way ANOVA: *F*_(3;13)_ = 6.296, *P* < 0.01, Newman–Keuls multiple comparison test: ^∗^*P* < 0.05, OGD vs. CTR; ^$^*P* < 0.05, OGD+PSB vs. OGD. CTR, *n* = 6; OGD, *n* = 5; OGD+PSB, *n* = 3; OGD+MRS, *n* = 3. **(F)** Statistical analysis: One-way ANOVA: *F*_(3,16)_ = 4.924, *P* < 0.02; Newman–Keuls multiple comparison test: ^∗^*P* < 0.05 OGD vs. CTR, ^#^*P* < 0.05 OGD+MRS vs. OGD,^$^*P* < 0.05 OGD+PSB vs. OGD. CTR, *n* = 6; OGD, *n* = 3; OGD+PSB, *n* = 4; OGD+MRS *n* = 4. All data in the graphs are expressed as mean ± SEM.

The extent of neuronal damage caused by 7 min OGD in stratum pyramidale of hippocampal CA1 was assessed by immunohistochemistry using the anti-NeuN antibody in control slices, in slices after 7 min OGD alone, and after 7 min OGD in the presence of 500 nM MRS1754 or 50 nM PSB603, both at 1 and 3 h after the end of OGD. Representative images of NeuN immunostaining in CA1 of slices collected 1 h after the end of OGD are shown in **Figures 5A–D**.

**Figures 5E,F** show the quantitative analyses of the area of NeuN^+^ immunofluorescence in CA1, which represents an index of the number of pyramidal neurons, 1 and 3 h after the end of OGD, respectively. The data demonstrate that NeuN^+^ CA1 pyramidal neurons significantly decreased both 1 h (**Figure 5E**) and 3 h (**Figure 5F**) after the end of 7 min OGD. Statistical analysis showed that 7 min OGD caused a statistically significant reduction of NeuN^+^ area at 1 h (-29.6%, ^∗^*P* < 0.05 vs. control slices) and at 3 h (-41%, ^∗^*P* < 0.05 vs. control slices). The time-course of the effect, indicating that the decrease of NeuN^+^ area was more pronounced at 3 h than at 1 h after the end of OGD, demonstrates that neuronal degeneration is an ongoing process at least at these time points.

The decrease of NeuN^+^ area in CA1 stratum pyramidale was completely antagonized by treatment with 50 nM PSB603 (-1% at 1 h and -14% at 3 h, ns vs. control slices). This effect was statistically significant vs. 7 min OGD slices both at 1 and 3 h after the end of OGD (^$^*P* < 0.05 vs. respective OGD). Treatment with 500 nM MRS1754 completely blocked the decrease of NeuN^+^ area in CA1 stratum pyramidale 3 h after the end of OGD (-7% vs. control slices, ns; ^#^*P* < 0.05 vs. OGD). MRS1754 had no effect 1 h after the end of OGD (-31.5% vs. control slices; ns vs. OGD). Therefore, antagonism of A_2B_ receptors blocked the neuronal damage induced by 7 min OGD up to 3 h after the end of the simil-ischemic insult. In the OGD slices treated either with MRS1754 or PSB603 that developed AD we found a partial reduction of neuronal damage at 1 h after the end of OGD (data not shown).

**FIGURE 6:**
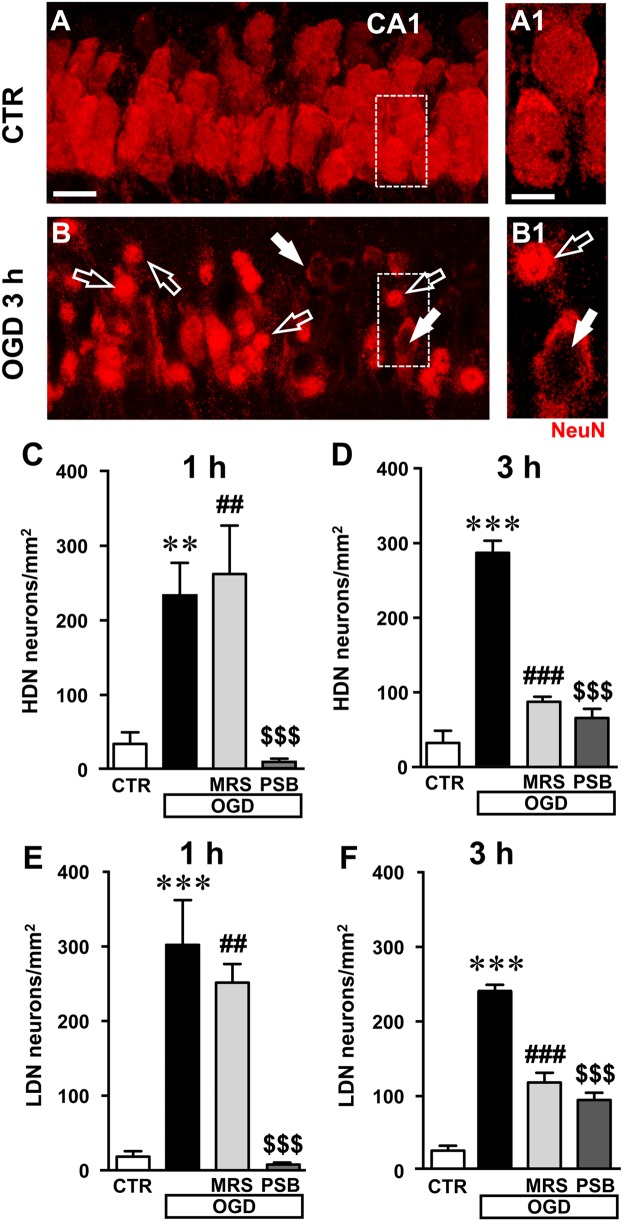
Analysis of damaged neurons in CA1 stratum pyramidale after the simil-ischemic insult. **(A–B1)** Representative images of NeuN^+^ immunofluorescence in the CA1 area of a control slice (CTR, **A,A1**), and of a slice harvested 3 h after the end of 7 min OGD (OGD, **B,B1**). **(A1–B1)** magnification of digital subslices of the framed areas in **A,B** (stacks of two consecutive z-scans taken at 2.1 μm depth inside the neurons, total thickness 0.6 μm). Note the presence of many HDN neurons (open arrows) and LDN neurons (white arrows) in CA1 stratum pyramidale after OGD **(B,B1)**. Scale bars: **A,B**: 25 μm; **A1,B1**: 10 μm. **(C,D)** Quantitative analyses of NeuN^+^ HDN neurons in CA1 stratum pyramidale 1 h **(C)** and 3 h **(D)** after the end of OGD. **(C)** One-way ANOVA: *F*_(3;12)_ = 11.32, *P* < 0.001. Newman–Keuls multiple comparison test: ^∗∗^*P* < 0.01, OGD vs. CTR; ^##^*P* < 0.01, OGD+MRS vs. CTR;^$$$^*P* < 0.001 OGD+PSB vs. OGD. CTR, *n* = 5; OGD, *n* = 5; OGD+PSB, *n* = 3; OGD+MRS, *n* = 3. **(D)** One-way ANOVA: *F*_(3;12)_ = 64.33, *P* < 0.001. Newman–Keuls multiple comparison test: ^∗∗∗^*P* < 0.001, OGD vs. CTR; ^###^*P* < 0.001, OGD+MRS vs. OGD;^$$$^*P* < 0.001 OGD+PSB vs. OGD. CTR, *n* = 5; OGD, *n* = 3; OGD+PSB, *n* = 4; OGD+MRS, *n* = 4. **(E–F)**: Quantitative analysis of NeuN^+^ LDN neurons in CA1 stratum pyramidale 1 h **(E)** and 3 h **(F)** after the end of OGD. **(E)** One-way ANOVA: *F*_(3;14)_ = 13.80, *P* < 0.001. Newman–Keuls multiple comparison test: ^∗∗∗^*P* < 0.01, OGD vs. CTR; ^##^*P* < 0.01, OGD+MRS vs. CTR;^$$$^*P* < 0.001 OGD+PSB vs. OGD. CTR, *n* = 6; OGD, *n* = 6; OGD+PSB, *n* = 3; OGD+MRS, *n* = 3. **(F)** One-way ANOVA: *F*_(3;12)_ = 69.77, *P* < 0.001. Newman–Keuls multiple comparison test: ^∗∗∗^*P* < 0.001, OGD vs. CTR; ^###^*P* < 0.001, OGD+MRS vs. OGD;^$$$^*P* < 0.001 OGD+PSB vs. OGD. CTR, *n* = 5; OGD, *n* = 3; OGD+PSB, *n* = 4; OGD+MRS, *n* = 4. All data in the graphs are expressed as mean ± SEM.

Closer examination of CA1 stratum pyramidale with confocal microscopy indicated the presence of many damaged neurons both 1 and 3 h after the end of 7 min OGD. The representative confocal z stacks in **Figures 5B**, **6B**, each obtained stacking 37 consecutive confocal z-scans (0.3 μm each, total thickness 11.1 μm) through the thickness of CA1, show that 3 h after the end of OGD the layout and morphology of CA1 pyramidal neurons was significantly different from that of the control slice (**Figure 6A**). **Figures 6A1,B1** are magnification of the framed areas in **Figures 6A,B**, and show stacks of two consecutive z-scans, 0.3 μm each, total thickness 0.6 μm, taken at 2.1 μm depth inside the neurons. It appears evident from panel **Figure 6B1** the altered morphology of pyramidal neurons after OGD, in comparison to those of the control slice shown in **Figure 6A1**. Indeed, in CA1 stratum pyramidale of OGD slices, both at 1 and 3 h after the end of OGD, we observed the presence of many neurons with nuclei that exhibit a highly condensed NeuN-positive nucleus and very faint NeuN cytoplasmic labeling (**Figures 6B,B1**, open arrows). We defined these neurons as High Density Nucleus neurons, “HDN neurons.” Furthermore, we observed many NeuN^+^ neurons that have lost the NeuN^+^ nuclear immunofluorescence, an index of damaged nuclei, while NeuN^+^ immunofluorescence persists in the cytoplasm (**Figures 6B,B1**, white arrows). We defined these neurons as Low Density Nucleus neurons, “LDN neurons.”

In order to better characterize this phenomenon, we performed the quantitative analysis of HDN and LDN neurons in control, 7 min OGD, 7 min OGD plus MRS1754 and 7 min OGD plus PSB603 slices at 1 and 3 h after the end of OGD. The results, presented in **Figures 6C,D**, show that HDN neurons increased significantly in 7 min OGD slices both at 1 h (+603% vs. control slices, ^∗∗^*P* < 0.01) and 3 h (+794% vs. control slices, ^∗∗∗^*P* < 0.001) after the end of OGD. The increase of damaged, HDN neurons in the CA1 area caused by the simil-ischemic insult was significantly blocked by treatment with 50 nM PSB603 at 1 and 3 h after the end of OGD (-97% at 1 h, and -77% at 3 h vs. 7 min OGD slices, both ^$$$^*P* < 0.001; ns vs. controls). Conversely, treatment with 500 nM MRS1754 significantly blocked the increase of HDN neurons only 3 h after the end of OGD (-70% vs. 7 min OGD slices, ^###^*P* < 0.001; ns vs. control slices), but not 1 h after the end of OGD (+12% vs. OGD slices, ns; ^##^ *P* < 0.01 vs. control slices).

Also, as shown by the representative images in **Figures 6B,B1**, we found many LDN neurons in stratum pyramidale 1 and 3 h after the end of 7 min OGD. As demonstrated by quantitative analysis (**Figures 6E,F**) LDN neurons in stratum pyramidale were significantly increased both 1 and 3 h after OGD, in comparison to control slices. The increase of LDN neurons, in comparison to control slices, was 1489% at 1 h (^∗∗∗^*P* < 0.01 vs. control slices) and 1033% at 3 h after the end of 7 min OGD (^∗∗∗^*P* < 0.01 vs. control slices). The increase of damaged, LDN neurons brought about by the simil-ischemic insult was significantly blocked by treatment with 50 nM PSB603 both at 1 and 3 h after the end of OGD (-98% at 1 h, and -62% at 3 h vs. OGD, both ^$$$^*P* < 0.001). Treatment with 500 nM MRS1754 significantly blocked the increase of LDN neurons only 3 h after the end of OGD (-52% vs. 7 min OGD,^###^*P* < 0.001), but not 1 h after the end of OGD (-17% vs. 7 min OGD, ns; ^##^*P* < 0.01 vs. controls). These data further confirm the efficacy of the two A_2B_ receptor antagonists, and particularly of PSB603, in reducing not only the electrophysiological effects but also the morphological modifications that OGD caused on CA1 pyramidal neurons, up to 3 h after the end of the ischemic-like insult.

### Analysis of Apoptotic Neurons in Stratum Pyramidale of CA1 1 and 3 h After 7 min OGD

**FIGURE 7:**
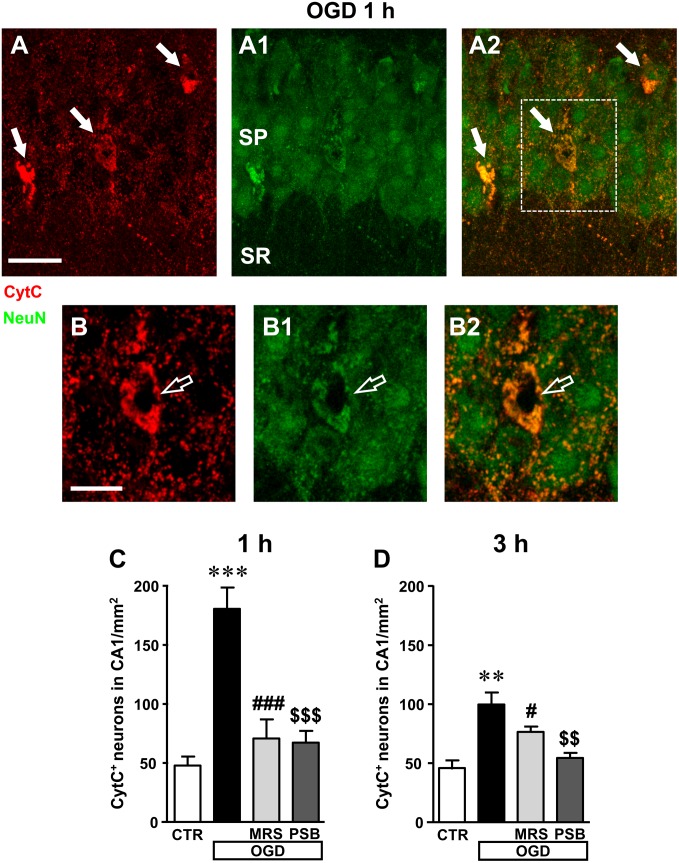
Analysis of CytocromeC^+^ (CytC^+^) neurons in CA1 stratum pyramidale after the simil-ischemic insult. **(A–A2)** Representative microphotographs, taken at the laser scanning confocal microscope, of apoptotic neurons labeled with anti-CytC antibody (**A**, red), of pyramidal neurons labeled with anti-NeuN antibody (**A1**, green) and the merge of the two previous images **(A2)**. NeuN^+^ and CytC^+^ apoptotic neurons in CA1 stratum pyramidale are indicated by the arrows (yellow-orange color in **A2**). Scale bar: 25 μm. **(B–B2)** Subslice of the framed area in **A2**, obtained stacking 17 consecutive confocal z-scans (5.1 μm total thickness), shown at higher magnification (2×). The open arrow shows an LDN apoptotic pyramidal neuron. Scale bar: 10 μm. **(C,D)** Quantitative analysis of NeuN^+^ and CytC^+^ neurons in CA1 stratum pyramidale at 1 h **(C)** and 3 h **(D)** after the end of 7 min OGD. Note the significant increase of CytC^+^ neurons both 1 and 3 h after the end of OGD. **(C)** Statistical analysis: One-way ANOVA: *F*_(3;11)_ = 18.40, *P* < 0.001, Newman–Keuls multiple comparison test: ^∗∗∗^*P* < 0.001, OGD vs. CTR; ^###^*P* < 0.001, OGD+MRS vs. OGD; ^$$$^*P* < 0.001, OGD+PSB vs. OGD. CTR, *n* = 4; OGD, *n* = 3; OGD+PSB, *n* = 4; OGD+MRS, *n* = 4. **(D)** Statistical analysis: One-way ANOVA: *F*_(3;11)_ = 11.41, *P* < 0.02, Newman–Keuls multiple comparison test: ^∗∗^*P* < 0.01, OGD vs. CTR; ^#^*P* < 0.05, OGD+MRS vs. OGD; ^$$^*P* < 0.01, OGD+PSB vs. OGD. CTR, *n* = 4; OGD, *n* = 3; OGD+PSB, *n* = 4; OGD+MRS, *n* = 4. All data in the graphs are expressed as mean ± SEM.

These data demonstrate that 7 min OGD can induce neuronal damage in CA1 stratum pyramidale, as evidenced by immunohistochemical analyses that highlight conformational modifications of pyramidal neurons that may subtend cell death. Therefore, we studied whether all the above-described effects and the decrease of neurons in CA1 stratum pyramidale might be caused by apoptosis. To this end, as an apoptosis marker we used CytC, a protein which, in physiological conditions, is found in mitochondria but in the most advanced stages of apoptosis is intensely and diffusely released in the cytoplasm, where it activates caspases (Kluck et al., 1997; Yang et al., 1997; Jiang and Wang, 2004; Suen et al., 2008) and can be used as a marker of apoptosis using immunohistochemical analysis (Martínez-Fábregas et al., 2014). Using a selective antibody, CytC can be visualized in apoptotic cells as an intense and diffuse cytoplasmic immunostaining, as shown by the white arrows in the representative confocal images of an OGD slice 1 h after the end of OGD (**Figures 7A–A2**). As shown in the confocal subslice of the framed area of **Figure 7A2**, obtained stacking 17 consecutive confocal z-scans through the CytC^+^ neuron (0.3 μm each, total thickness 5.1 μm), it is evident that the CytC^+^ positive neuron is a LDN neuron (**Figures 7B–B2**, open arrow), thus demonstrating that LDN neurons are apoptotic.

From the quantitative analysis of CytC^+^ neurons in CA1 stratum pyramidale, we demonstrated that both 1 and 3 h after the end of 7 min OGD many CA1 pyramidal neurons were apoptotic (**Figures 7C,D**). The increase was statistically significant in comparison to control slices both at 1 h (+277% vs. control slices, ^∗∗∗^*P* < 0.001) and at 3 h (+107% vs. control slices, ^∗∗^*P* < 0.01) after OGD. These data indicate that in CA1 area, already after 1 h from the end of OGD, neurons had clear signs of apoptotic processes. In the presence of MRS1754 or PSB603, there was a significant reduction of CytC immunostaining, both at 1 and 3 h after the end of OGD, showing that antagonism of A_2B_ receptors significantly reduced neuronal death by apoptosis at both times investigated. Indeed, treatment with MRS1754 decreased apoptotic neurons by 61% at 1 h (^###^*P* < 0.001 vs. 7 min OGD; ns vs. control slices) and by 33% at 3 h (^#^*P* < 0.05 vs. 7 min OGD; ns vs. control slices), in comparison to OGD slices. Treatment with PSB603 decreased apoptotic neurons by 63% (^$$$^*P* < 0.001 vs. 7 min OGD; ns vs. control slices) and by 46% (^$$^*P* < 0.001 vs. 7 min OGD; ns vs. control slices) in comparison to OGD slices. In the OGD slices treated either with MRS1754 or PSB603 that developed AD the number of HDN and LDN neurons were partially decreased in comparison to OGD slices (data not shown).

These data indicate that in the CA1 area already 1 h after the end of OGD, when there was still no recovery of neurotransmission, neurons showed obvious signs of apoptosis. These data demonstrate that antagonism of A_2B_ receptors brought about significant protection against neuron degeneration.

### Analysis of Phospho-mTOR in Area CA1 of the Hippocampus 1 and 3 h After 7 min OGD

**FIGURE 8:**
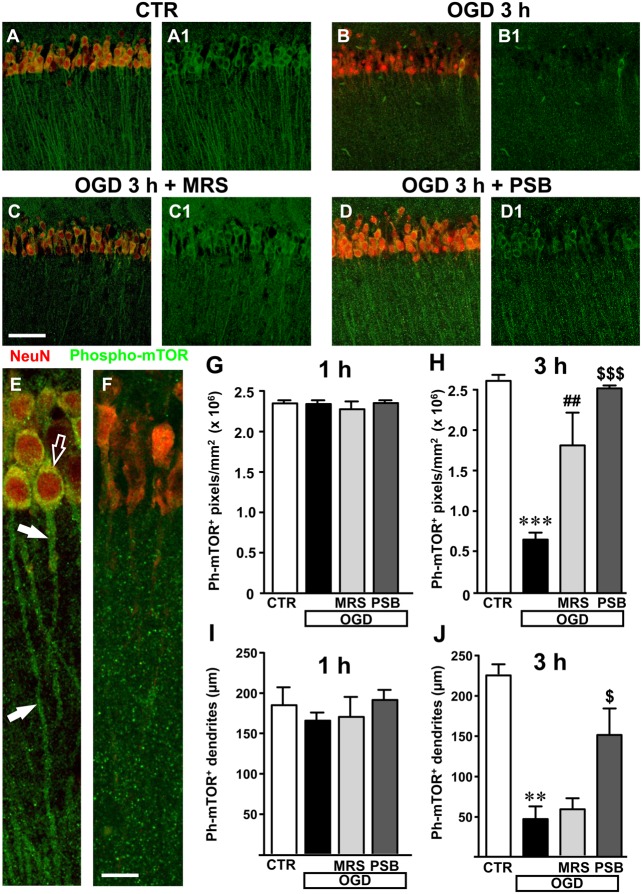
mTOR activation in CA1 stratum pyramidale and stratum radiatum after the simil-ischemic insult. Representative microphotographs, taken at the laser scanning confocal microscope, showing immunolabelling with anti-NeuN antibody (red) and anti-phospho-mTOR antibody (green) of a control slice **(A,A1)**, a slice harvested 3 h after 7 min OGD **(B,B1)**, a slice treated with MRS1754 and harvested 3 h after 7 min OGD **(C,C1)**, and a slice treated with PSB603 and harvested 3 h after 7 min OGD **(D,D1)**. Scale bar: 75 μm. **(E,F)** Digital subslices of a control slice **(E)** and a slice collected 3 h after 7 min OGD **(F)** immunostained for phospho-mTOR (green) and NeuN (red). The open arrow shows the presence of activated mTOR in the cell body and arrows in the dendrites of pyramidal neurons in the control slice **(E)**. **(G,H)** Quantitative analysis of activated mTOR in CA1 stratum pyramidale in the different experimental conditions. Each column represents the mTOR^+^ immunofluorescent area calculated using the ImageJ program (number of pixels above a reference, fixed threshold). **(G)** No difference among the four experimental groups, was found 1 h after the end of 7 min OGD. Statistical analysis: One-way ANOVA: *F*_(3;11)_ = 0.4563, *P* > 0.05, ns. **(H)** Slices harvested 3 h after the end of 7 min OGD. Note the significant decrease of activated mTOR in CA1 pyramidal neurons 3 h after the end of OGD. Both MRS1754 and PSB603 significantly blocked this effect. Statistical analysis: One-way ANOVA: *F*_(3;10)_ = 26.99, *P* < 0.001, Newman–Keuls multiple comparison test: ^∗∗∗^*P* < 0.001, OGD vs. CTR; ^##^*P* < 0.01, OGD+MRS vs. OGD; ^$$$^*P* < 0.001, OGD +PSB vs. OGD. CTR, *n* = 4; OGD, *n* = 3; OGD+PSB, *n* = 3; OGD+MRS, *n* = 4. All data are expressed as mean ± SEM. **(I,J)** Quantitative analysis of phospho-mTOR^+^ dendrites in CA1 stratum radiatum in the different experimental conditions. **(I)** Length of phospho-mTOR^+^ dendrites in CA1 stratum radiatum 1 h after the end of 7 min OGD. No difference among the four experimental groups was observed. Statistical analysis: One-way ANOVA: *F*_(3;11)_ = 0.7143, *P* > 0.05, ns. **(J)** Length of mTOR^+^ dendrites in CA1 stratum radiatum 3 h after the end of 7 min OGD. Note the significant decrease of activated mTOR in dendrites 3 h after the end of OGD. PSB603 significantly blocked this effect. Statistical analysis: One-way ANOVA: *F*_(3;9)_ = 12.38, *P* < 0.02, Newman–Keuls multiple comparison test: ^∗∗^*P* < 0.01, OGD vs. CTR; ^$^*P* < 0.05, OGD+PSB vs. OGD. CTR, *n* = 3; OGD, *n* = 3; OGD+PSB, *n* = 3; OGD+MRS, *n* = 4. All data in the graphs are expressed as mean ± SEM.

We used a selective antibody for phospho-(Ser244)-mTOR, the activated form of mTOR, to investigate whether mTOR activation might be modified in our experimental conditions (**Figures 8A–D1**). Representative qualitative images of mTOR activation in cell bodies and dendrites of CA1 pyramidal neurons in a control slice are shown in **Figure 8A1** (green). Neurons were also immunolabelled with anti-NeuN antibody (red). The merge of the immunofluorescence (yellow-orange) in a control slice is shown in **Figure 8A**. It is evident from the images that activated mTOR is present in CA1 pyramidal neurons in basal, control conditions where it is localized both in the cell body and in neuronal apical dendritic tree spanning throughout the stratum radiatum. The simil-ischemic condition caused a significant decrease of mTOR activation 3 h after the end of OGD, as shown in the representative image of **Figures 8B,B1**. This effect is more evident in **Figures 8E,F**, that represent digital subslices obtained stacking nine consecutive confocal z-scans throughout the neuronal cell bodies (0.3 μm each, total thickness 2.7 μm) of control and OGD slices. The images clearly show that in control conditions phospho-mTOR was present both in the cell body (**Figure 8E**, open arrow) and in the dendrites (**Figure 8E**, white arrows), while 3 h after 7 min OGD activation of mTOR decreased both in cell body and dendrites (**Figure 8F**). Quantitative analysis showed that in slices harvested 1 h after the end of 7 min OGD, no significant modification of activated mTOR immunostaining was present in the neuronal cell body (**Figure 8G**) or in the apical dendrites of CA1 pyramidal neurons in any of the groups investigated (**Figure 8I**). On the contrary, in slices harvested 3 h after the end of 7 min OGD, we found highly significant decrease of activated mTOR immunostaining in the cytoplasm and dendrites of CA1 pyramidal neurons (**Figures 8H,J**). Indeed, statistical analysis, shown in **Figure 8H**, demonstrates that 3 h after the end of 7 min OGD there was a statistically significant reduction of activated mTOR immunostaining in the cytoplasm of CA1 pyramidal neurons (-74.8%, ^∗∗∗^*P* < 0.001 vs. control slices, **Figure 8H**) in comparison to control slices. As shown in **Figure 8H**, treatment with 50 nM PSB603 blocked this effect (-4% vs. control slices, ns, ^$$$^*P* < 0.001 vs. 7 min OGD), while treatment with 500 nM MRS1754 partially, but still significantly attenuated this effect (-31% vs. controls, ns, ^##^*P* < 0.01 vs. 7 min OGD).

We used, as a determinant of mTOR activation in the dendrites, the analysis of the length of phospho-mTOR positive dendrites, as reported in the methods. The results shown in **Figure 8I** reveal that mTOR activation was not statistically significant among the four experimental groups 1 h after the end of 7 min OGD. However, in the slices collected 3 h after the end of 7 min OGD we found a significant decrease of mTOR positive dendrites in the stratum radiatum of the CA1 area (**Figure 8J**). From the statistical analysis we demonstrated a significant decrease of mTOR immunopositive dendrites in CA1 stratum radiatum of 7 min OGD slices 3 h after the end of OGD (-80% vs. controls, ^∗∗^*P* < 0.01, **Figure 8J**). The selective antagonist MRS1754, did not significantly modify this effect, while treatment with PSB603 partially, but significantly, reversed this effect (+226% vs. 7 min OGD,^$^*P* < 0.05). These data demonstrate that OGD significantly decreased mTOR activation and that the selective antagonism selective antagonism of A_2B_ receptors significantly reduced this impairment, a further indication of prevention of neuronal degeneration by blockade of this receptor.

### Analysis of Astrocytes in CA1 Stratum Radiatum After 7 min OGD

**FIGURE 9:**
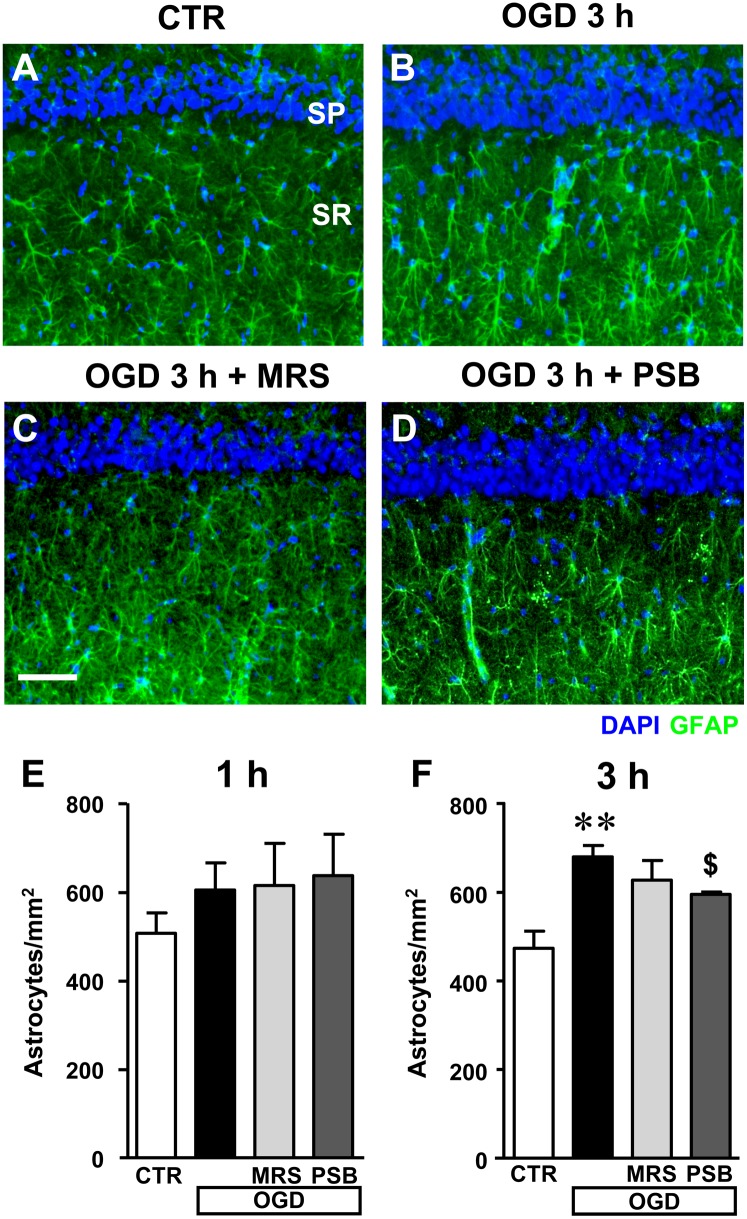
Quantitative analysis of astrocytes in the CA1 area in the different experimental conditions after the simil-ischemic insult. **(A–D)** Representative microphotographs, taken at the epifluorescence microscope, of astrocytes immunolabelled with anti-GFAP antibodies in the stratum radiatum (green) of a control **(A)**, OGD **(B)**, OGD plus MRS1754 **(C)**, and OGD plus PSB603 **(D)** slice. Scale bar: 50 μm. **(E,F)** Quantitative analysis of astrocytes in the stratum radiatum of CA1 in control, OGD, OGD plus MRS1754, and OGD plus PSB603 slices at 1 h **(E)** and 3 h **(F)** after 7 min OGD. **(E)** No significant differences among the four experimental groups analyzed was found. Statistical analysis: One-way ANOVA: *F*_(3;18)_ = 0.877, *P* > 0.05, ns. CTR, *n* = 8; OGD, *n* = 7; OGD+PSB, *n* = 3; OGD+MRS, *n* = 4. **(F)** Statistical analysis: One-way ANOVA: *F*_(3;15)_ = 6.734, *P* < 0.01, Newman–Keuls multiple comparison test: ^∗∗^*P* < 0.01, OGD vs. CTR; ^$^*P* < 0.05, OGD+PSB vs. OGD. CTR, *n* = 7; OGD, *n* = 4; OGD+PSB, *n* = 4; OGD+MRS, *n* = 4. All data in the graphs are expressed as mean ± SEM.

Astrocytes were labeled with the anti-GFAP antibody and quantified in the stratum radiatum of CA1 hippocampus in the four experimental conditions: in control slices, in slices after 7 min OGD alone, and after 7 min OGD in the presence of 500 nM MRS1754 or 50 nM PSB603, both at 1 and 3 h after the end of OGD, as shown in the representative microphotographs in **Figures 9A–D**, taken at 3 h after the end of OGD.

In the stratum radiatum of slices harvested 1 h after the end of 7 min OGD we found a slight, not significant increase of astrocytes (**Figure 9E**, +19%, ns vs. controls), which became significant at 3 h after the end of 7 min OGD (**Figure 9F**, +43% vs. control slices, ^∗∗^*P* < 0.01). Both A_2B_ receptor antagonists, partially but significantly, reduced the increase of astrocytes caused by the simil-ischemic conditions. MRS1754 decreased the number of astrocytes by 10% (ns vs. OGD), while PSB603 by 13% (^$^*P* < 0.05 vs. OGD).

Quantitative analysis of total microglia did not reveal statistically significant modifications in the different experimental conditions both at 1 and 3 h after the end of 7 min OGD (data not shown).

### Neurodegeneration of CA1 Pyramidal Neurons Induced by Glutamate Was Not Prevented by Adenosine A_2B_ Receptor Antagonists

**FIGURE 10:**
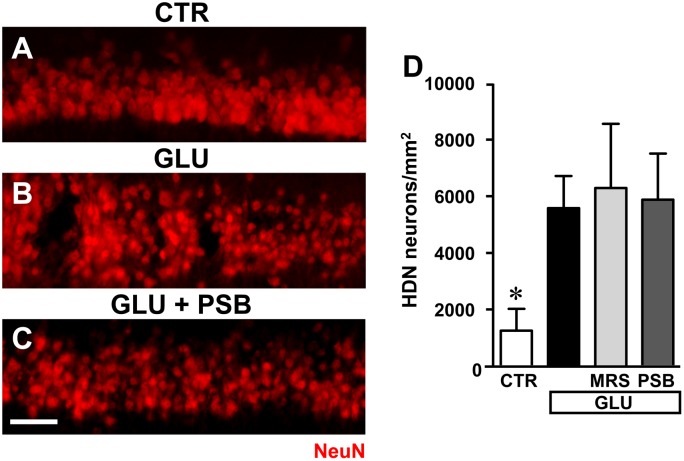
Evaluation of glutamate induced neurotoxicity in CA1 stratum pyramidale in the different experimental conditions. **(A–C)** Representative microphotographs, taken at the epifluorescence microscope, of CA1 pyramidal neurons immunolabelled with anti-NeuN antibodies in a control slice **(A)**, a slice treated with glutamate (GLU, **B**), a slice treated with glutamate plus PSB603 (GLU+ PSB, **C**) slice. Scale bar: 50 μm. **(D)** Quantitative analyses of NeuN^+^ HDN neurons in the four experimental groups 3 h after the end of drug incubation. Statistical analysis: One-way ANOVA: *F*_(3;15)_ = 3.313, *P* < 0.05, Newman–Keuls multiple comparison test: ^∗^*P* < 0.05, vs. all other groups. CTR, *n* = 6; GLU, *n* = 6; GLU+PSB, *n* = 3; GLU+MRS, *n* = 4.

In order to have an insight into the mechanism of A_2B_ receptor antagonism-induced neuroprotection, we verified whether MRS1754 and PSB603 might protect CA1 pyramidal neurons from the well-known neurodegenerative effects caused by glutamate exposure. We incubated the hippocampal slices *in vitro* with 100 μM glutamate for 10 min and verified the effect of MRS1754 and PSB603 on glutamate-induced cell death (**Figures 10A–D**).

Administration of 100 μM glutamate for 10 min caused significant damage to pyramidal neurons at 3 h after the end of incubation, evidenced by the significant increase of HDN neurons in hippocampal CA1, as shown in the representative image presented in **Figure 10B**. Quantitative analysis (**Figure 10D**) demonstrated that the increase of HDN neurons was statistically significant in comparison to control slices, and that neither MRS1754 nor PSB603 protected CA1 pyramidal neurons from the excitotoxic effect of glutamate (^∗^*P* < 0.05 vs. all other groups).

## Discussion

The putative protective role of adenosine A_2B_ receptors in cerebral ischemia was studied in the CA1 region of hippocampal slices under oxygen-glucose deprivation, an experimental condition that mimics, albeit with the limits of *in vitro* methodology, the most common causes of cerebral ischemia, such as vessel occlusion. *In vitro* slices give a partial view of the physiology of the brain because of the absence of an intact vascular system and the altered tridimensional microenvironment. These alterations involve not only neurons but also glia, and more generally the physiology of the neurovascular unit formed by astrocytes, pericytes, microglia, neurons, and the extracellular matrix (Holloway and Gavins, 2016). Nevertheless, the *in vitro* systems have many benefits such as the opportunity to obtain highly valuable information in terms of the time-course of the electrophysiological events, changes in membrane potential (AD), changes in synaptic transmission and morphological and biochemical changes in neurons and glia. Our results confirm that in the CA1 region of rat hippocampus, the application of a 7 min OGD episode induced the appearance of AD which was followed by irreversible synaptic damage and neurodegeneration of CA1 pyramidal neurons (Pugliese et al., 2003, 2006, 2009; Coppi et al., 2007; Traini et al., 2011). We now demonstrate that these events are accompanied by neurodegeneration of CA1 pyramidal neurons, with reduction of neuronal density and significant increase of apoptotic neurons. For the first time we demonstrated here that antagonism of A_2B_ receptors using the selective ligands MRS1754 or PSB603, applied before, during and after OGD, prevented or delayed the appearance of AD, and prevented the irreversible loss of neurotransmission induced by 7 min OGD. Furthermore, we demonstrated that the selective blockade of A_2B_ receptors during a prolonged (30 min) OGD insult delays the appearance of AD indicating an extension of the time window between the start of the insult and the appearance of excitotoxic damage. Adenosine A_2B_ receptor antagonism also counteracted the reduction of neuronal density in CA1 stratum pyramidale and decreased apoptosis mechanisms at least up to 3 h after the end of the insult. Both A_2B_ receptor antagonists did not protect CA1 neurons from neurodegeneration induced by glutamate application, indicating that the antagonistic effect is upstream of glutamate release.

The hippocampus, and particularly CA1 stratum pyramidale, is one of the most vulnerable brain regions to ischemic damage. We used the acute rat hippocampal slice preparation which allows measurements of synaptic transmission with good spatial and temporal resolution. In the early phases, hypoxia/ischemia is known to induce a massive increase of extracellular glutamate levels which trigger hyperactivation of glutamate receptors, production of reactive oxygen species, pathological increase of intracellular Ca^2+^, rapid decrease in ATP reserves and activation of various proteolytic enzymes (Káradóttir et al., 2005; Al-Majed et al., 2006; Kovacs et al., 2006). In hippocampal slices, a severe OGD insult as that applied in the present experiments (7 min) elicits the appearance of AD within the OGD period and is invariably followed by irreversible loss of neurotransmission (Frenguelli et al., 2007; Pugliese et al., 2007, 2009), an index of cell suffering, damage to neurons and to the surrounding tissue (Somjen, 2001). AD is caused by the sudden increase of extracellular K^+^ and by the contemporary explosive rise in glutamate extracellular concentration (Somjen, 2001). Contemporarily to the extracellular increase of glutamate, the extracellular concentration of adenosine significantly increases, as demonstrated both in *in vivo* and *in vitro* experiments (Latini and Pedata, 2001). After 5 min OGD, adenosine reaches an extracellular concentration of 30 μM in hippocampal slices (Latini et al., 1999; Pearson et al., 2006). At such high concentration adenosine can stimulate all receptor subtypes, including the A_2B_ receptor, which exhibits affinity for adenosine with an EC_50_ in the range of 5–20 μM, lower than all other subtypes (Fredholm et al., 2011). For this reason, it is possible that activation of A_2B_ receptors occurs mainly during pathological conditions, such as inflammation, hypoxia, trauma, and ischemia (Fredholm et al., 2001).

Our data show that A_2B_ receptor antagonists, by preventing or delaying the onset of AD, prevent the irreversible loss of neurotransmission induced by 7 min OGD allowing complete recovery of synaptic potentials. We showed for the first time a partial recovery of neurotransmission was also observed in a group of hippocampal slices, treated with A_2B_ receptor antagonists, that developed AD immediately after reoxygenation. This delay of AD appearance might account for the partial recovery of neurotransmission observed in these slices. The occurrence of AD after the end of OGD period is a peculiar characteristic that we observed in our hippocampal preparation. We envisage that when the AD appears during the reoxygenation period, similarly to the phenomenon of spreading depression (Somjen, 2001), neurons are less damaged, and they can partially recover their electrical activity. Thus, even in those slices treated with the A_2B_ receptor antagonists in which AD takes place, this event is less harmful to neuronal viability. This is a substantial difference from A_2A_ receptor antagonist-mediated neuroprotection during a 7 min OGD insult. Indeed, fEPSP recovery was never observed in those few slices undergoing AD in the presence of the A_2A_ receptor blocker ZM241385, as previously published (Pugliese et al., 2009).

As to the mechanism by which A_2B_ receptor antagonists protect from hypoxia/ischemia, recent studies by Gonçalves et al. (2015) have demonstrated that in mouse hippocampus A_2B_ receptors are expressed on glutamatergic terminals anatomically comparable to those from which our recordings were performed. Their selective stimulation counteracts the predominant A_1_ receptor-mediated inhibition of synaptic transmission. We confirmed this assumption by performing PPF experiments in rat hippocampal slices in which the A_2B_ receptor agonist BAY606583 was able to reduce PPF ratio, which is known to be caused by increased glutamate release at presynaptic level. This effect is counteracted not only by the A_2B_ receptor antagonists MRS1754 and PSB603 but also by the A_1_ receptor antagonist DPCPX. As already hypothesized (Moriyama and Sitkovsky, 2010; Gonçalves et al., 2015), this result may be related to the existence of an A_1_/A_2B_ receptor heterodimer in the CA1 hippocampal region. On these bases, our purpose was to study the possible involvement of A_1_ receptors in the neuroprotective effects elicited by the two A_2B_ receptor antagonists during OGD. In accordance to data reported by Canals et al. (2008) in a model of chemical penumbra produced by a mitochondrial gliotoxin in the hippocampus *in vitro*, we would have expected conservation of synaptic transmission during the first min of OGD and acceleration of AD appearance. In our conditions, a similar response was observed only in a limited number of slices during 7 min OGD. Instead, in most of the slices we demonstrated that DPCPX induced neuroprotection during OGD, delaying AD appearance. This unexpected result may be due to a different response of A_1_ receptors during OGD in our experimental conditions. Our data could also be explained considering the results obtained by Sperlágh et al. (2007) who demonstrated that DPCPX decreases glutamate release from hippocampal slices subjected to OGD and that this effect is mimicked and occluded by the A_2A_ receptor antagonist ZM241385. The same Authors hypothesize that DPCPX, even at low nanomolar concentrations, would directly bind to A_2A_ receptors during severe ischemia (in accordance with our previously published results, Pugliese et al., 2009). The rational for this assumption is that the A_2A_ receptor agonist CGS21680 displays two distinct binding sites in the hippocampus: a “typical” (striatal-like) binding site which is displayed by DPCPX only at high (submicromolar) concentrations, and an “atypical” binding site, which shows high affinity for DPCPX (Johansson and Fredholm, 1995; Cunha et al., 1996). On these bases, we can hypothesize that, in our OGD experiments, DPCPX binds to this “atypical” binding site (i.e., an A_1_–A_2A_ receptor heterodimer) thus decreasing glutamate outflow and protecting hippocampal slices from OGD insults. Furthermore, when overstimulated such as during ischemia, A_1_ receptors undergo desensitization (Siniscalchi et al., 1999). This phenomenon can be further increased by A_2B_ receptors activation, triggering a vicious circle in which the beneficial effect of A_1_ receptor stimulation is overcome by the noxious effect of A_2B_ receptors activation (Gonçalves et al., 2015) as already suggested for A_2A_ adenosine receptors (Pugliese et al., 2009). Further mechanistic studies suggest that the A_2A_ receptor, when stimulated, facilitates A_2B_ receptor externalization from the endoplasmic reticulum to the plasma membrane, possibly increasing the formation of the A_2A_–A_2B_ dimer (Moriyama and Sitkovsky, 2010). All these results taken together may explain the deleterious activity of adenosine A_2B_ receptor stimulation during an ischemic insult, and the protective effect of A_2B_ receptor antagonists in this condition. Finally, observation that the A_2B_ receptor antagonists did not protect CA1 neurons from neurodegeneration induced by direct glutamate application, confirms that the mechanism underlying their protection against ischemia-induced neurodegeneration is exerted at adenosine receptors that, by the abovementioned mechanisms, regulate extracellular glutamate release. Alternatively, since OGD is above all a problem of efficient energy recovery, the demonstration that A_2B_ receptors control astrocytic and neuronal glycogen metabolism (Magistretti et al., 1986; Allaman et al., 2003) and glucose utilization by hippocampal slices (Lemos et al., 2015) may suggest an additional effect of these receptors on metabolic activity during OGD.

Severe OGD increased apoptosis and damaged CA1 pyramidal neurons at 1 and 3 h after the end of the ischemic insult. Immunohistochemistry showed that CA1 pyramidal neurons had significant morphological changes, with increased density of nuclei (HDN neurons), karyorrhexis (LDN neurons) and possibly nuclear fragmentation, as evidenced by the significantly higher number of LDN neurons and cell death after OGD. These results are in agreement with those found by Ünal-Çevik et al. (2004) in the cerebral cortex of the rat after mild ischemia. Pyknosis is typical of apoptotic cells (Elmore, 2007) and may precede karyorrhexis. We demonstrated that LDN neurons, being highly positive for CytC, were undergoing apoptosis. It has been demonstrated that CytC released into the cytosol binds to apoptotic protease activating factor-1, which leads to activation of caspase-9 which is important in neuronal cell death following ischemia (Kluck et al., 1997; Yang et al., 1997; Love, 2003; Jiang and Wang, 2004; Suen et al., 2008; Lana et al., 2014, 2016, 2017a,b; Martínez-Fábregas et al., 2014). In turn, caspase-9 is activated by high glutamate levels, as occurs during ischemia (Li et al., 2009). As reported in the literature, activation of mTOR, which has multiple roles in cells among which local protein synthesis at the dendritic and spine level (Frey and Morris, 1997; Tsokas et al., 2007; Thoreen et al., 2012), can be modified in ischemic conditions (Dennis et al., 2001; Laplante and Sabatini, 2012). As already reported (Gegelashvili et al., 2001; Maragakis and Rothstein, 2004), the decrease of mTOR activation may be secondary to the excitotoxic mechanisms evoked by massive increase of glutamate during OGD, which is known to be an important component of neuronal injury *in vitro* (Newell et al., 1995). The participation of decreased mTOR activation in OGD-induced neuronal damage is supported by our results showing decreased activation of mTOR both in the cell body and dendrites of CA1 neurons 3 h after the end of OGD.

Within the limits of the *in vitro* model and the alteration of the neurovascular unit and of neuro glia interplay, we found interesting effects on astrocytic responses. Indeed, astrocytes proliferation, possibly caused, among other stimuli, by increased release of glutamate, is one of the early events that takes place after acute focal CNS damage (Burda and Sofroniew, 2014). In accordance to our previous results (Pugliese et al., 2009), we found evidence of significant, although limited, astrocytic proliferation in CA1 stratum radiatum already 3 h after the end of OGD, possibly caused by increased glutamate release. A_2B_ receptor antagonism significantly prevented all the above neuronal and astrocytic modifications, sparing neurons from the degenerative effects caused by the simil-ischemic conditions, and reducing astrocytes proliferation. CA1 pyramidal neurons treated with the A_2B_ receptor antagonists had a similar morphology to those of control slices, had neither increased nor decreased nuclear density, did not undergo apoptosis, and had activated mTOR levels similar to those of controls.

The similar effects obtained using two different A_2B_ receptor antagonists strengthen the hypothesis that the A_2B_ receptor is involved in the mechanisms of cerebral ischemia. Nevertheless, MRS1754 seems to have lower efficiency than PSB603 on some of the parameters investigated. It is possible that the two drugs act with a different time-course or that PSB603 is more efficacious than MRS1754 in this model.

In summary, our data demonstrate that antagonists of adenosine A_2B_ receptors protect the CA1 area of the hippocampus from an acute damage induced by severe hypoxic/ischemic conditions. The mechanism likely resides in protection from the acute increase of glutamate extracellular concentrations and consequent excitotoxicity. It is worth noticing that since A_2B_ receptors have low affinity for the endogenous ligand adenosine, they are activated only at high extracellular adenosine concentrations that can be reached under pathological conditions such as ischemia, thus representing a selective target (Popoli and Pepponi, 2012).

## Author Contributions

MG and AP designed the research. FU, DL, DN, IF, LG, ID, and FC performed the experiments. FU, DL, EC, MG and AP analyzed the data. DL, MG, EC, AP and FP interpreted the results and the experiments. DL, MG, FU, AP and IF prepared the figures. MG and AP drafted the manuscript. DL, MG, FU, FP, AP and EC edited and revised the manuscript. All authors read and approved the final version of the manuscript.

## Conflict of Interest Statement

The authors declare that the research was conducted in the absence of any commercial or financial relationships that could be construed as a potential conflict of interest.

## Footnotes

1 http://rsb.info.nih.gov/ij

## References

[B1] Allaman I. Lengacher S. Magistretti P. J. Pellerin L. (2003). A2B receptor activation promotes glycogen synthesis in astrocytes through modulation of gene expression. *Am. J. Physiol. Cell Physiol.* 284 C696–C704. 10.1152/ajpcell.00202.2002 12421692

[B2] Allard D. Turcotte M. Stagg J. (2017). Targeting A2 adenosine receptors in cancer. *Immunol. Cell Biol.* 95 333–339. 10.1038/icb.2017.8 28174424

[B3] Al-Majed A. A. Sayed-Ahmed M. M. Al-Omar F. A. Al-Yahya A. A. Aleisa A. M. Al-Shabanah O. A. (2006). Carnitine esters prevent oxidative stress damage and energy depletion following transient forebrain ischaemia in the rat hippocampus. *Clin. Exp. Pharmacol. Physiol.* 33 725–733. 10.1111/j.1440-1681.2006.04425.x 16895547

[B4] Anderson W. W. Collingridge G. L. (2001). The LTP Program: a data acquisition program for on-line analysis of long-term potentiation and other synaptic events. *J. Neurosci. Methods* 108 71–83. 10.1016/S0165-0270(01)00374-0 11459620

[B5] Burda J. E. Sofroniew M. V. (2014). Reactive gliosis and the multicellular response to CNS damage and disease. *Neuron* 81 229–248. 10.1016/j.neuron.2013.12.034 24462092 PMC3984950

[B6] Canals S. Larrosa B. Pintor J. Mena M. A. Herreras O. (2008). Metabolic challenge to glia activates an adenosine-mediated safety mechanism that promotes neuronal survival by delaying the onset of spreading depression waves. *J. Cereb. Blood Flow Metab.* 28 1835–1844. 10.1038/jcbfm.2008.71 18612316

[B7] Cerbai F. Lana D. Nosi D. Petkova-Kirova P. Zecchi S. Brothers H. M. (2012). The neuron-astrocyte-microglia triad in normal brain ageing and in a model of neuroinflammation in the rat hippocampus. *PLoS One* 7:e45250. 10.1371/journal.pone.0045250 23028880 PMC3445467

[B8] Chandrasekera P. C. McIntosh V. J. Cao F. X. Lasley R. D. (2010). Differential effects of adenosine A2a and A2b receptors on cardiac contractility. *Am. J. Physiol. Heart Circ. Physiol.* 299 H2082–H2089. 10.1152/ajpheart.00511.2010 20935155 PMC3006297

[B9] Chen J. F. Sonsalla P. Pedata F. Melani A. Domenici M. R. Popoli P. (2007). Adenosine A2A receptors and brain injury: broad spectrum of neuroprotection, multi-faced actions and “fine tuning” modulation. *Prog. Neurobiol.* 83 310–331. 10.1016/j.pneurobio.2007.09.002 18023959

[B10] Choi D. W. (1992). Excitotoxic cell death. *J. Neurobiol.* 23 1261–1276. 10.1002/neu.480230915 1361523

[B11] Coppi E. Pugliese A. M. Stephan H. Müller C. E. Pedata F. (2007). Role of P2 purinergic receptors in synaptic transmission under normoxic and ischaemic conditions in the CA1 region of rat hippocampal slices. *Purinergic Signal.* 3 203–219. 10.1007/s11302-006-9049-4 18404434 PMC2096646

[B12] Csóka B. Németh Z. H. Selmeczy Z. Koscsó B. Pacher P. Vizi E. S. (2007). Role of A(2A) adenosine receptors in regulation of opsonized *E. coli-*induced macrophage function. *Purinergic Signal.* 3 447–452. 10.1007/s11302-007-9075-x 18404457 PMC2072923

[B13] Cunha R. A. Johansson B. Constantino M. D. Sebastião A. M. Fredholm B. B. (1996). Evidence for high-affinity binding sites for the adenosine A2A receptor agonist [3H] CGS 21680 in the rat hippocampus and cerebral cortex that are different from striatal A2A receptors. *Naunyn Schmiedebergs Arch. Pharmacol.* 353 261–271. 10.1007/BF00168627 8692280

[B14] Dennis P. B. Jaeschke A. Saitoh M. Fowler B. Kozma S. C. Thomas G. (2001). Mammalian TOR: a homeostatic ATP sensor. *Science* 294 1102–1105. 10.1126/science.1063518 11691993

[B15] Dirnagl U. (2012). Pathobiology of injury after stroke: the neurovascular unit and beyond. *Ann. N. Y. Acad. Sci.* 1268 21–25. 10.1111/j.1749-6632.2012.06691.x 22994217

[B16] Dixon A. K. Gubitz A. K. Sirinathsinghji D. J. Richardson P. J. Freeman T. C. (1996). Tissue distribution of adenosine receptor mRNAs in the rat. *Br. J. Pharmacol.* 118 1461–1468. 10.1111/j.1476-5381.1996.tb15561.x 8832073 PMC1909676

[B17] Elmore S. (2007). Apoptosis: a review of programmed cell death. *Toxicol. Pathol.* 35 495–516. 10.1080/01926230701320337 17562483 PMC2117903

[B18] Farkas E. Pratt R. Sengpiel F. Obrenovitch T. P. (2008). Direct, live imaging of cortical spreading depression and anoxic depolarisation using a fluorescent, voltage-sensitive dye. *J. Cereb. Blood Flow Metab.* 28 251–262. 10.1038/sj.jcbfm.9600569 17971792 PMC2653938

[B19] Feoktistov I. Polosa R. Holgate S. T. Biaggioni I. (1998). Adenosine A2B receptors: a novel therapeutic target in asthma? *Trends Pharmacol. Sci.* 19 148–153. 9612090 10.1016/s0165-6147(98)01179-1

[B20] Fowler J. C. (1992). Escape from inhibition of synaptic transmission during in vitro hypoxia and hypoglycemia in the hippocampus. *Brain Res.* 573 169–173. 10.1016/0006-8993(92)90128-V 1315606

[B21] Fredholm B. B. IJzerman A. P. Jacobson K. A. Linden J. Müller C. E. (2011). International union of basic and clinical pharmacology. LXXXI. Nomenclature and classification of adenosine receptors–an update. *Pharmacol. Rev.* 63 1–34. 10.1124/pr.110.003285 21303899 PMC3061413

[B22] Fredholm B. B. Irenius E. Kull B. Schulte G. (2001). Comparison of the potency of adenosine as an agonist at human adenosine receptors expressed in Chinese hamster ovary cells. *Biochem. Pharmacol.* 61 443–448. 10.1016/S0006-2952(00)00570-0 11226378

[B23] Frenguelli B. G. Wigmore G. Llaudet E. Dale N. (2007). Temporal and mechanistic dissociation of ATP and adenosine release during ischaemia in the mammalian hippocampus. *J. Neurochem.* 101 1400–1413. 10.1111/j.1471-4159.2007.04425.x 17459147 PMC1920548

[B24] Frey U. Morris R. G. (1997). Synaptic tagging and long-term potentiation. *Nature* 385 533–536. 10.1038/385533a0 9020359

[B25] Gegelashvili G. Robinson M. B. Trotti D. Rauen T. (2001). Regulation of glutamate transporters in health and disease. *Prog. Brain Res.* 132 267–286. 10.1016/S0079-6123(01)32082-4 11544995

[B26] Gonçalves F. Q. Pires J. Pliassova A. Beleza R. Lemos C. Marques J. M. (2015). Adenosine A2b receptors control A1 receptor-mediated inhibition of synaptic transmission in the mouse hippocampus. *Eur. J. Neurosci.* 41 878–888. 10.1111/ejn.12851 25704806

[B27] Herreras O. Somjen G. G. (1993). Propagation of spreading depression among dendrites and somata of the same cell population. *Brain Res.* 610 276–282. 10.1016/0006-8993(93)91411-K 8100471

[B28] Holloway P. M. Gavins F. N. (2016). Modeling ischemic stroke in vitro: status quo and future perspectives. *Stroke* 47 561–569. 10.1161/STROKEAHA.115.011932 26742797 PMC4729600

[B29] Jarvis C. R. Anderson T. R. Andrew R. D. (2001). Anoxic depolarization mediates acute damage independent of glutamate in neocortical brain slices. *Cereb. Cortex* 11 249–259. 10.1093/cercor/11.3.249 11230096

[B30] Jiang X. Wang X. (2004). Cytochrome C-mediated apoptosis. *Annu. Rev. Biochem.* 73 87–106. 10.1146/annurev.biochem.73.011303.073706 15189137

[B31] Johansson B. Fredholm B. B. (1995). Further characterization of the binding of the adenosine receptor agonist [3H]CGS 21680 to rat brain using autoradiography. *Neuropharmacology* 34 393–403. 10.1016/0028-3908(95)00009-U 7566470

[B32] Káradóttir R. Cavelier P. Bergersen L. H. Attwell D. (2005). NMDA receptors are expressed in oligodendrocytes and activated in ischaemia. *Nature* 438 1162–1166. 10.1038/nature04302 16372011 PMC1416283

[B33] Kluck R. M. Bossy-Wetzel E. Green D. R. Newmeyer D. D. (1997). The release of cytochrome c from mitochondria: a primary site for Bcl-2 regulation of apoptosis. *Science* 275 1132–1136. 10.1126/science.275.5303.1132 9027315

[B34] Kolachala V. Ruble B. Vijay-Kumar M. Wang L. Mwangi S. Figler H. (2008). Blockade of adenosine A2B receptors ameliorates murine colitis. *Br. J. Pharmacol.* 155 127–137. 10.1038/bjp.2008.227 18536750 PMC2440087

[B35] Koroleva V. I. Bures J. (1996). The use of spreading depression waves for acute and long-term monitoring of the penumbra zone of focal ischemic damage in rats. *Proc. Natl. Acad. Sci. U.S.A.* 93 3710–3714. 10.1073/pnas.93.8.3710 8623001 PMC39677

[B36] Kovacs K. Toth A. Deres P. Kalai T. Hideg K. Gallyas F. Jr. (2006). Critical role of PI3-kinase/Akt activation in the PARP inhibitor induced heart function recovery during ischemia-reperfusion. *Biochem. Pharmacol.* 71 441–452. 10.1016/j.bcp.2005.05.036 16337154

[B37] Lana D. Cerbai F. Di Russo J. Boscaro F. Giannetti A. Petkova-Kirova P. (2013). Hippocampal long term memory: effect of the cholinergic system on local protein synthesis. *Neurobiol. Learn. Mem.* 106 246–257. 10.1016/j.nlm.2013.09.013 24076274

[B38] Lana D. Iovino L. Nosi D. Wenk G. L. Giovannini M. G. (2016). The neuron-astrocyte-microglia triad involvement in neuroinflammaging mechanisms in the CA3 hippocampus of memory-impaired aged rats. *Exp. Gerontol.* 83 71–88. 10.1016/j.exger.2016.07.011 27466072

[B39] Lana D. Melani A. Pugliese A. M. Cipriani S. Nosi D. Pedata F. (2014). The neuron-astrocyte-microglia triad in a rat model of chronic cerebral hypoperfusion: protective effect of dipyridamole. *Front. Aging Neurosci.* 6:322. 10.3389/fnagi.2014.00322 25505884 PMC4245920

[B40] Lana D. Ugolini F. Melani A. Nosi D. Pedata F. Giovannini M. G. (2017a). The neuron-astrocyte-microglia triad in CA3 after chronic cerebral hypoperfusion in the rat: protective effect of dipyridamole. *Exp. Gerontol.* 96 46–62. 10.1016/j.exger.2017.06.006 28606482

[B41] Lana D. Ugolini F. Nosi D. Wenk G. L. Giovannini M. G. (2017b). Alterations in the interplay between neurons, astrocytes and microglia in the rat dentate gyrus in experimental models of neurodegeneration. *Front. Aging Neurosci.* 9:296. 10.3389/fnagi.2017.00296 28955220 PMC5601988

[B42] Laplante M. Sabatini D. M. (2012). mTOR signaling in growth control and disease. *Cell* 149 274–293. 10.1016/j.cell.2012.03.017 22500797 PMC3331679

[B43] Latini S. Bordoni F. Corradetti R. Pepeu G. Pedata F. (1998). Temporal correlation between adenosine outflow and synaptic potential inhibition in rat hippocampal slices during ischemia-like conditions. *Brain Res.* 794 325–328. 10.1016/S0006-8993(98)00304-7 9622666

[B44] Latini S. Bordoni F. Corradetti R. Pepeu G. Pedata F. (1999). Effect of A2A adenosine receptor stimulation and antagonism on synaptic depression induced by in vitro ischaemia in rat hippocampal slices. *Br. J. Pharmacol.* 128 1035–1044. 10.1038/sj.bjp.0702888 10556941 PMC1571729

[B45] Latini S. Pedata F. (2001). Adenosine in the central nervous system: release mechanisms and extracellular concentrations. *J. Neurochem.* 79 463–484. 10.1046/j.1471-4159.2001.00607.x 11701750

[B46] Lemos C. Pinheiro B. S. Beleza R. O. Marques J. M. Rodrigues R. J. Cunha R. A. (2015). Adenosine A2B receptor activation stimulates glucose uptake in the mouse forebrain. *Purinergic Signal.* 1 561–569. 10.1007/s11302-015-9474-3 26446689 PMC4648789

[B47] Li S. Q. Zhang Y. Tang D. B. (2009). Possible mechanisms of Cyclosporin A ameliorated the ischemic microenvironment and inhibited mitochondria stress in tree shrews’ hippocampus. *Pathophysiology* 16 279–284. 10.1016/j.pathophys.2009.02.014 19303263

[B48] Love S. (2003). Apoptosis and brain ischaemia. *Prog. Neuropsychopharmacol. Biol. Psychiatry* 27 267–282. 12657366 10.1016/S0278-5846(03)00022-8

[B49] Magistretti P. J. Hof P. R. Martin J. L. (1986). Adenosine stimulates glycogenolysis in mouse cerebral cortex: a possible coupling mechanism between neuronal activity and energy metabolism. *J. Neurosci.* 6 2558–2562. 10.1523/JNEUROSCI.06-09-02558.1986 3018195 PMC6568677

[B50] Maragakis N. J. Rothstein J. D. (2004). Glutamate transporters: animal models to neurologic diseases. *Neurobiol. Dis.* 15 461–473. 10.1016/j.nbd.2003.12.007 15056453

[B51] Martínez-Fábregas J. Díaz-Moreno I. González-Arzola K. Janocha S. Navarro J. A. Hervás M. (2014). Structural and functional analysis of novel human cytochrome C targets in apoptosis. *Mol. Cell. Proteomics* 13 1439–1456. 10.1074/mcp.M113.034322 24643968 PMC4047465

[B52] Melani A. Pantoni L. Corsi C. Bianchi L. Monopoli A. Bertorelli R. (1999). Striatal outflow of adenosine, excitatory amino acids, gamma-aminobutyric acid, and taurine in awake freely moving rats after middle cerebral artery occlusion: correlations with neurological deficit and histopathological damage. *Stroke* 30 2448–2454. 10.1161/01.STR.30.11.2448 10548683

[B53] Merighi S. Borea P. A. Gessi S. (2015). Adenosine receptors and diabetes: Focus on the A(2B) adenosine receptor subtype. *Pharmacol. Res.* 99 229–236. 10.1016/j.phrs.2015.06.015 26142494

[B54] Moriyama K. Sitkovsky M. V. (2010). Adenosine A2A receptor is involved in cell surface expression of A2B receptor. *J. Biol. Chem.* 285 39271–39288. 10.1074/jbc.M109.098293 20926384 PMC2998132

[B55] Newell D. W. Barth A. Papermaster V. Malouf A. T. (1995). Glutamate and non-glutamate receptor mediated toxicity caused by oxygen, and glucose deprivation in organotypic hippocampal cultures. *J. Neurosci.* 15 7702–7711. 10.1523/JNEUROSCI.15-11-07702.1995 7472521 PMC6578073

[B56] Pearson T. Damian K. Lynas R. E. Frenguelli B. G. (2006). Sustained elevation of extracellular adenosine and activation of A1 receptors underlie the post-ischaemic inhibition of neuronal function in rat hippocampus in vitro. *J. Neurochem.* 97 1357–1368. 10.1111/j.1471-4159.2006.03823.x 16696848

[B57] Pedata F. Dettori I. Coppi E. Melani A. Fusco I. Corradetti R. (2016). Purinergic signalling in brain ischemia. *Neuropharmacology* 104 105–130. 10.1016/j.neuropharm.2015.11.007 26581499

[B58] Pedata F. Latini S. Pugliese A. M. Pepeu G. (1993). Investigations into the adenosine outflow from hippocampal slices evoked by ischemia-like conditions. *J. Neurochem.* 61 284–289. 10.1111/j.1471-4159.1993.tb03566.x 8515275

[B59] Pedata F. Pugliese A. M. Coppi E. Dettori I. Maraula G. Cellai L. (2014). Adenosine A2A receptors modulate acute injury and neuroinflammation in brain ischemia. *Mediators Inflamm.* 2014:805198. 10.1155/2014/805198 25165414 PMC4138795

[B60] Perez-Buira S. Barrachina M. Rodriguez A. Albasanz J. L. Martín M. Ferrer I. (2007). Expression levels of adenosine receptors in hippocampus and frontal cortex in argyrophilic grain disease. *Neurosci. Lett.* 423 194–199. 10.1016/j.neulet.2007.06.049 17707587

[B61] Popoli P. Pepponi R. (2012). Potential therapeutic relevance of adenosine A2B and A2A receptors in the central nervous system. *CNS Neurol. Disord. Drug Targets* 11 664–674. 10.2174/187152712803581100 22963436

[B62] Pugliese A. M. Coppi E. Spalluto G. Corradetti R. Pedata F. (2006). A3 adenosine receptor antagonists delay irreversible synaptic failure caused by oxygen and glucose deprivation in the rat CA1 hippocampus in vitro. *Br. J. Pharmacol.* 147 524–532. 10.1038/sj.bjp.0706646 16415905 PMC1616978

[B63] Pugliese A. M. Coppi E. Volpini R. Cristalli G. Corradetti R. Jeong L. S. (2007). Role of adenosine A3 receptors on CA1 hippocampal neurotransmission during oxygen-glucose deprivation episodes of different duration. *Biochem. Pharmacol.* 74 768–779. 10.1016/j.bcp.2007.06.003 17626785 PMC2000832

[B64] Pugliese A. M. Latini S. Corradetti R. Pedata F. (2003). Brief, repeated, oxygen-glucose deprivation episodes protect neurotransmission from a longer ischemic episode in the in vitro hippocampus: role of adenosine receptors. *Br. J. Pharmacol.* 140 305–314. 10.1038/sj.bjp.0705442 12970092 PMC1574038

[B65] Pugliese A. M. Traini C. Cipriani S. Gianfriddo M. Mello T. Giovannini M. G. (2009). The adenosine A2A receptor antagonist ZM241385 enhances neuronal survival after oxygen-glucose deprivation in rat CA1 hippocampal slices. *Br. J. Pharmacol.* 157 818–830. 10.1111/j.1476-5381.2009.00218.x 19422385 PMC2721266

[B66] Siniscalchi A. Rodi D. Gessi S. Campi F. Borea P. A. (1999). Early changes in adenosine A1 receptors in cerebral cortex slices submitted to in vitro ischemia. *Neurochem. Int.* 34 517–522. 10.1016/S0197-0186(99)00028-5 10402227

[B67] Somjen G. G. (2001). Mechanisms of spreading depression and hypoxic spreading depression-like depolarization. *Physiol. Rev.* 81 1065–1096. 10.1152/physrev.2001.81.3.1065 11427692

[B68] Sperlágh B. Zsilla G. Baranyi M. Illes P. Vizi E. S. (2007). Purinergic modulation of glutamate release under ischemic-like conditions in the hippocampus. *Neuroscience* 149 99–111. 10.1016/j.neuroscience.2007.07.035 17850981

[B69] Suen D. F. Norris K. L. Youle R. J. (2008). Mitochondrial dynamics and apoptosis. *Genes Dev.* 22 1577–1590. 10.1101/gad.1658508 18559474 PMC2732420

[B70] Tanaka E. Yamamoto S. Kudo Y. Mihara S. Higashi H. (1997). Mechanisms underlying the rapid depolarization produced by deprivation of oxygen and glucose in rat hippocampal CA1 neurons in vitro. *J. Neurophysiol.* 78 891–902. 10.1152/jn.1997.78.2.891 9307122

[B71] Thoreen C. C. Chantranupong L. Keys H. R. Wang T. Gray N. S. Sabatini D. M. (2012). A unifying model for mTORC1-mediated regulation of mRNA translation. *Nature* 485 109–113. 10.1038/nature11083 22552098 PMC3347774

[B72] Traini C. Pedata F. Cipriani S. Mello T. Galli A. Giovannini M. G. (2011). P2 receptor antagonists prevent synaptic failure and extracellular signal-regulated kinase 1/2 activation induced by oxygen and glucose deprivation in rat CA1 hippocampus in vitro. *Eur. J. Neurosci.* 33 2203–2215. 10.1111/j.1460-9568.2011.07667.x 21453436

[B73] Tsokas P. Ma T. Iyengar R. Landau E. M. Blitzer R. D. (2007). Mitogen-activated protein kinase upregulates the dendritic translation machinery in long-term potentiation by controlling the mammalian target of rapamycin pathway. *J. Neurosci.* 27 5885–5894. 10.1523/JNEUROSCI.4548-06.2007 17537959 PMC6672260

[B74] Ugolini F. Lana D. Fusco I. Coppi E. Dettori I. Gaviano L. (2017). “The selective antagonism of adenosine A2B receptors prevents synaptic and neuronal damage induced by oxygen and glucose deprivation in CA1 rat hippocampus,” in *Proceedings of the Neuroscience 47th Annual Meeting*, Washington, DC.

[B75] Ünal-Çevik I. Kilinç M. Gürsoy-Ozdemir Y. Gurer G. Dalkara T. (2004). Loss of NeuN immunoreactivity after cerebral ischemia does not indicate neuronal cell loss: a cautionary note. *Brain Res.* 1015 169–174. 10.1016/j.brainres.2004.04.032 15223381

[B76] Yamamoto S. Tanaka E. Shoji Y. Kudo Y. Inokuchi H. Higashi H. (1997). Factors that reverse the persistent depolarization produced by deprivation of oxygen and glucose in rat hippocampal CA1 neurons in vitro. *J. Neurophysiol.* 78 903–911. 10.1152/jn.1997.78.2.903 9307123

[B77] Yang J. Liu X. Bhalla K. Kim C. N. Ibrado A. M. Cai J. (1997). Prevention of apoptosis by Bcl-2: release of cytochrome c from mitochondria blocked. *Science* 275 1129–1132. 10.1126/science.275.5303.1129 9027314

