# Supplementary material for: The Selective Antagonism of Adenosine A2B Receptors Reduces the Synaptic Failure and Neuronal Death Induced by Oxygen and Glucose Deprivation in Rat CA1 Hippocampus in Vitro
Source: Front Pharmacol. 2018 Apr 24;9:399. doi: 10.3389/fphar.2018.00399 (PMC5928446; doi:10.3389/fphar.2018.00399)
Supplement: Supplementary file 1 [file Data_Sheet_1.DOCX]

**Supplementary Image 1**: The selective block of adenosine A_2B_ receptors allowed a recovery of synaptic potentials up to 24 h after the end of 7 min OGD. **A-C**: fEPSPs were recorded before (pre-OGD) and at different times after the end of OGD in untreated OGD slice (A), in 500 nM MRS1754-treated OGD slice (B) and in 50 nM PSB603-treated OGD slice (C). Each trace, taken from a typical experiment, represents the average of two consecutive fEPSPs. Note that after OGD in one untreated OGD slice only the afferent volley was recorded. In control conditions (inset: no OGD) a stable fEPSP can be recorded for up to 24 h after slice preparation. Calibration: 1 mV, 5 ms.
