# Supplementary material for: The Selective Antagonism of Adenosine A2B Receptors Reduces the Synaptic Failure and Neuronal Death Induced by Oxygen and Glucose Deprivation in Rat CA1 Hippocampus in Vitro
Source: Front Pharmacol. 2018 Apr 24;9:399. doi: 10.3389/fphar.2018.00399 (PMC5928446; doi:10.3389/fphar.2018.00399)
Supplement: Supplementary file 2 [file Data_Sheet_2.DOCX]

**Supplementary Image 2**: Effect of DPCPX, a selective adenosine A_1_ receptor antagonist, on OGD-induced damage in rat hippocampal slices. **A-B:** AD was recorded as a negative d.c. shift in response to 7 min OGD in the absence (A, n=4) or in the presence (B, n=6) of 500 nM DPCPX. Note that the A_1_ adenosine receptor antagonist prevented the appearance of AD in 2 out of 6 slices. **C:** The graph shows the time course of the effect of 7 min OGD on fEPSP amplitude (mean±SEM) in untreated (n=4) OGD slices and in 500 nM DPCPX- (n=6) treated OGD slices. Note that, after reperfusion in normal oxygenated standard solution, a recovery of fEPSP was found only in DPCPX -treated OGD slices in which AD did not occur. In the presence of the A_1_ receptor antagonist the time course of fEPSP depression during OGD was delayed in comparison to corresponding times in the absence of the drug. **D**: The graph shows the time course of the effect of 30 min OGD on fEPSP amplitude in OGD-untreated slice (n=12) and 100 nM DPCPX-treated slices (n=13). **E:** Each column represents the mean±SEM of AD latency recorded in hippocampal slices during 30 min OGD in the absence, in the presence of 100 nM DPCPX or 50 µM D-AP5, a potent, selective NMDA antagonist. AD was measured from the beginning of the OGD insult. Note that AD was significantly delayed in the presence of DPCPX or D-AP5. *P<0.05, **P<0.01, One-way ANOVA followed by Newman-Keuls Multiple comparison test, both vs OGD.
