# Supplementary figures and images for: The Selective Antagonism of Adenosine A2B Receptors Reduces the Synaptic Failure and Neuronal Death Induced by Oxygen and Glucose Deprivation in Rat CA1 Hippocampus in Vitro
Source: Front Pharmacol. 2018 Apr 24;9:399. doi: 10.3389/fphar.2018.00399 (PMC5928446; doi:10.3389/fphar.2018.00399)

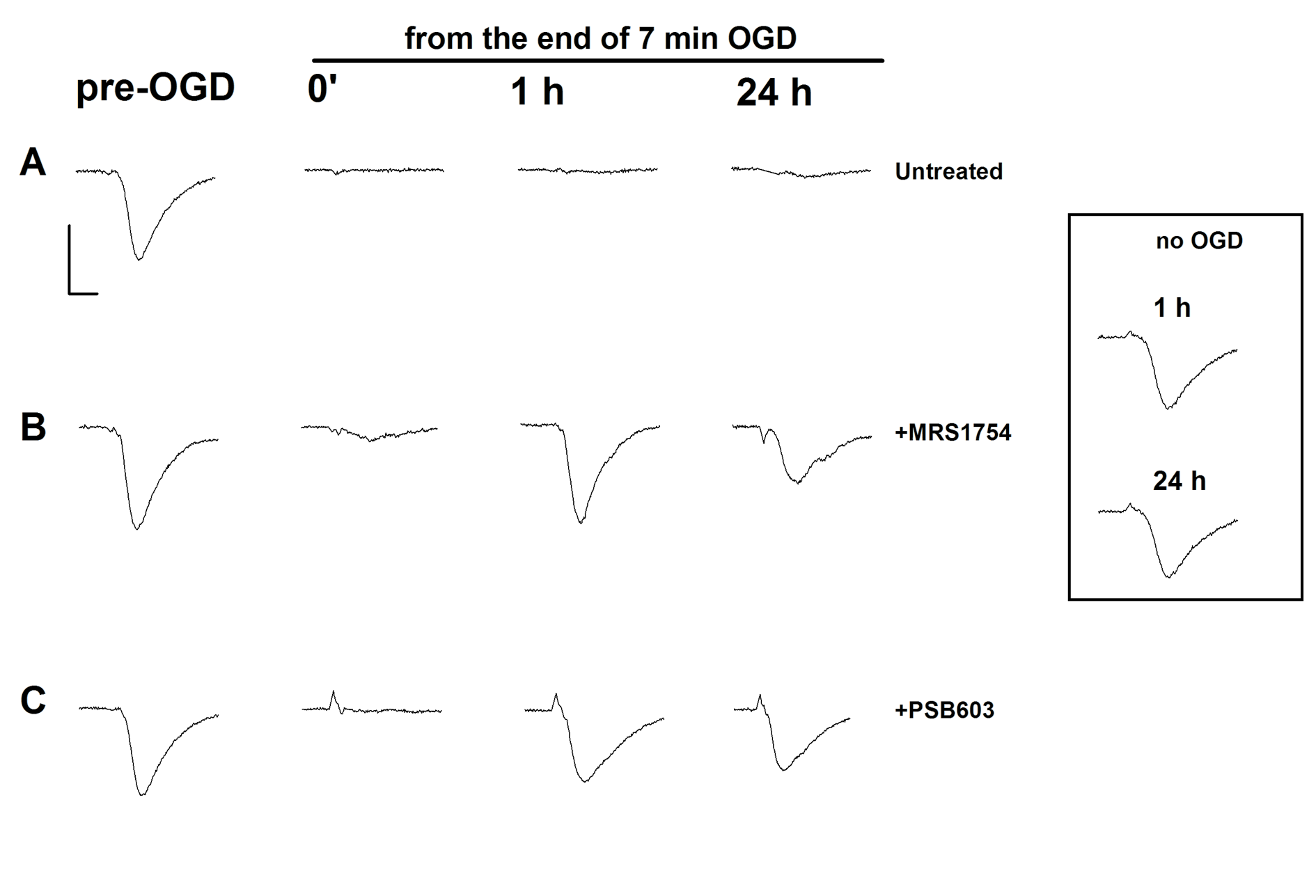

Supplement: Supplementary file 3 [file Image_1.TIF]

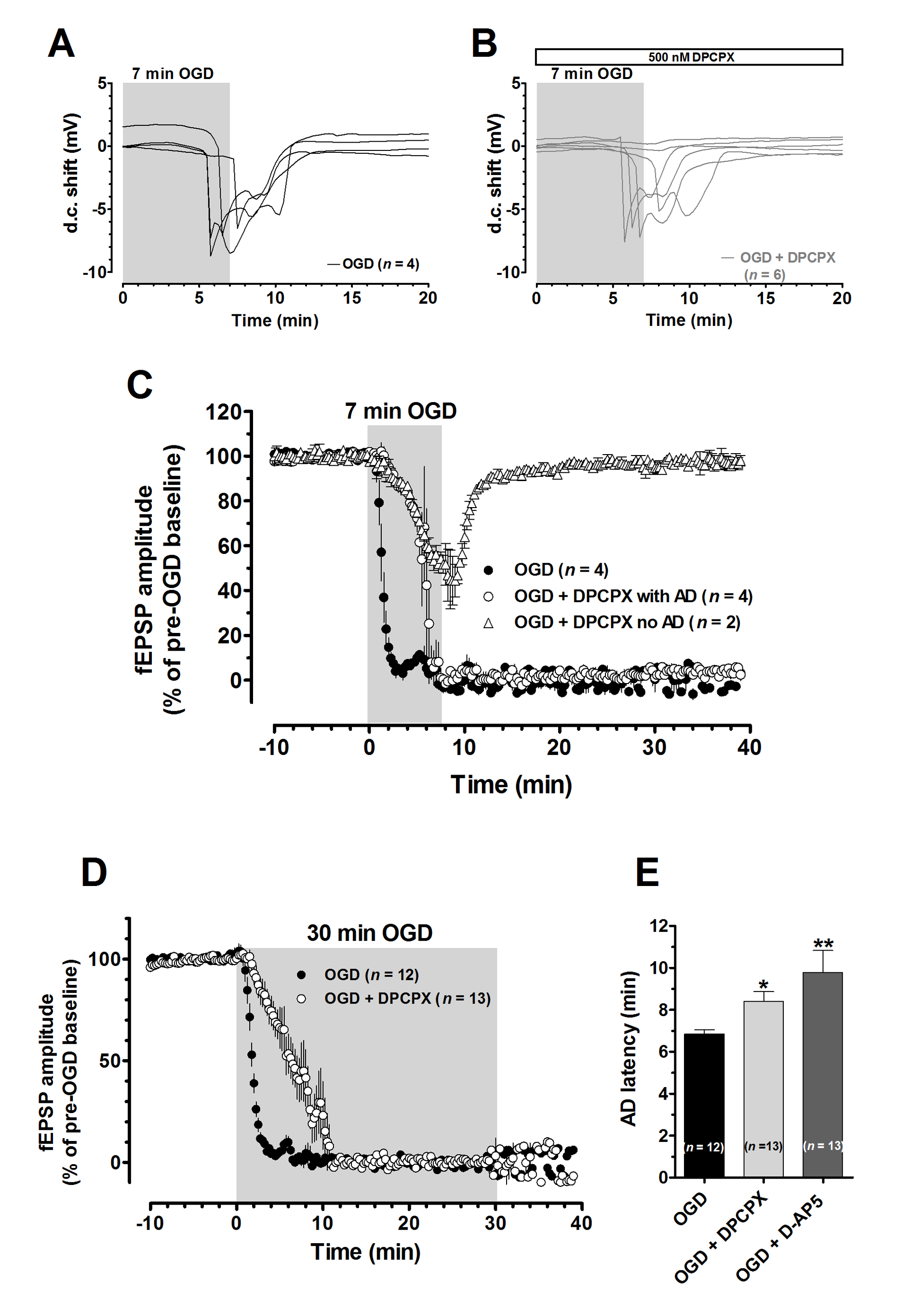

Supplement: Supplementary file 4 [file Image_2.TIF]
